# Meta-analysis of DNA methylation biomarkers in hepatocellular carcinoma

**DOI:** 10.18632/oncotarget.13221

**Published:** 2016-11-08

**Authors:** Cheng Zhang, Jinyun Li, Tao Huang, Shiwei Duan, Dongjun Dai, Danjie Jiang, Xinbing Sui, Da Li, Yidan Chen, Fei Ding, Changxin Huang, Gongying Chen, Kaifeng Wang

**Affiliations:** ^1^ Department of Medical Oncology, The Affiliated Hospital of Hangzhou Normal University, Hangzhou, Zhejiang, China; ^2^ Zhejiang Provincial Key Laboratory of Pathophysiology, School of Medicine, Ningbo University, Ningbo, Zhejiang, China; ^3^ Department of Medical Oncology, Sir Run Run Shaw Hospital, Zhejiang University, Hangzhou, Zhejiang, China

**Keywords:** meta-analysis, DNA methylation, biomarker, hepatocellular carcinoma

## Abstract

DNA methylation is an epigenetic mechanism in the pathogenesis of hepatocellular carcinoma (HCC). Here, we conducted a systematic meta-analysis to evaluate the contribution of DNA methylation to the risk of HCC. A total of 2109 publications were initially retrieved from PubMed, Web of Science, Cochrane Library, Embase, CNKI and Wanfang literature database. After a four-step filtration, we harvested 144 case-control articles in the meta-analysis. Our results revealed that 24 genes (carcinoma tissues *vs* adjacent tissues), 17 genes (carcinoma tissues *vs* normal tissues) and six genes (carcinoma serums *vs* normal serums) were significantly hypermethylated in HCC. Subgroup meta-analysis by geographical populations showed that six genes (carcinoma tissues *vs* adjacent tissues) and four genes (carcinoma tissues *vs* normal tissues) were significantly hypermethylated in HCC. Our meta-analysis identified the correlations between a number of aberrant methylated genes (*p16*, *RASSF1A*, *GSTP1*, *p14*, *CDH1*, *APC*, *RUNX3*, *SOCS1*, *p15*, *MGMT*, *SFRP1*, *WIF1*, *PRDM2*, *DAPK1*, *RARβ*, *hMLH1*, *p73*, *DLC1*, *p53*, *SPINT2*, *OPCML* and *WT1*) and HCC. Aberrant DNA methylation might become useful biomarkers for the prediction and diagnosis of HCC.

## INTRODUCTION

Hepatocellular carcinoma (HCC) is one of the most frequent malignancies and the sixth leading cause of cancer-related deaths in the United States [1]. The development of HCC is caused by the interaction of environmental, genetic and epigenetic factors [2, 3], such as aflatoxin exposure, alcohol consumption, hepatitis virus infection and familial tendency [4–6]. In addition, epigenetic modification is involved in HCC pathogenesis [3], and aberrant DNA methylation is the primary mediator of epigenetic changes in HCC [7].

Methylation of CpG islands in the gene promoters is recognized as a common epigenetic mechanism of transcriptional regulations [8, 9], and it has been shown to have a relation to the occurrence and development of several types of carcinomas [10–14]. Aberrant DNA methylation of the gene promoters may become promising biomarkers for the early diagnosis of diseases [15, 16]. Several studies have suggested that aberrant methylation of multiple tumor suppressor genes may contribute to the pathogenesis of HCC and epigenetic inactivation provides a prognostic value for determining the risk for the development of HCC [17, 18].

Aberrant patterns of DNA methylation in HCC can be useful for the prediction of cancer risk. We conduct a comprehensive meta-analysis based on the accumulation of the HCC association studies on DNA methylation to provide molecular clues of the potential diagnostic biomarkers with aberrant DNA methylation in HCC.

## RESULTS

**Figure 1:**
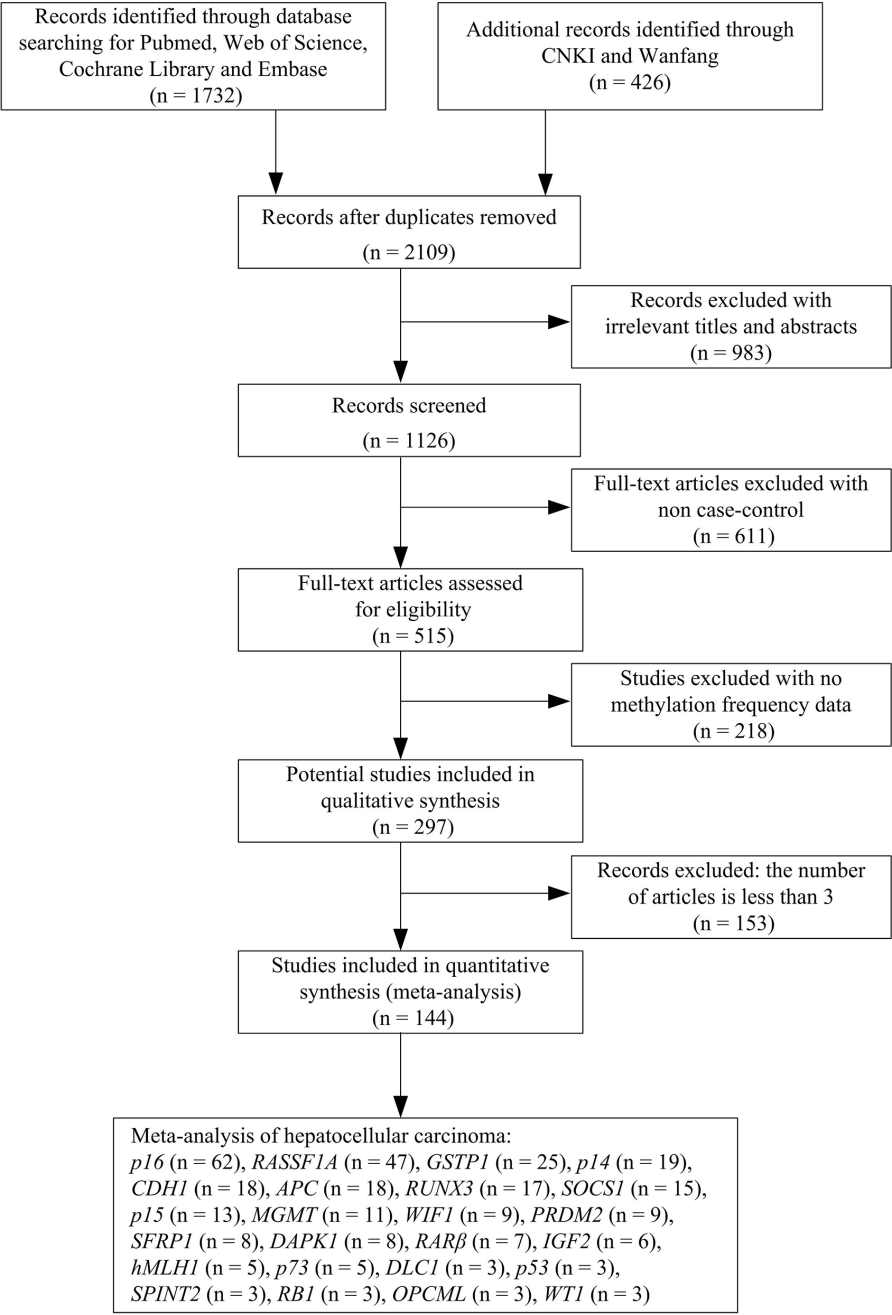
Flow diagram of the stepwise selection from the relevant studies

A total of 2109 studies were identified by our initial research using the keywords “hepatocellular carcinoma or hepatocarcinoma or primary liver cancer or HCC or hepatic carcinoma or liver tumor” and “DNA methylation” from PubMed, Web of Science, Cochrane Library, Embase, CNKI and Wanfang literature database. After a series of selection procedure shown in Figure 1, we excluded 983 irrelevant studies, 611 non-case control studies, 218 studies without methylation frequency data and 153 studies less than 3 articles. Thus, a total of 144 eligible studies were included in the current meta-analysis (Supplementary Document S1). The 144 case-control studies published from 2000 to 2016 included 6523 HCC tumor tissues, 5498 adjacent tissues, 689 normal tissues, 2044 HCC serums and 1371 normal serums within 24 genes. Among these 24 genes, the meta-analysis of *p16*, *RASSF1A*, *CDH1*, *RUNX3* and *GSTP1* genes methylation was performed between HCC tumor tissues *vs* adjacent tissues, HCC tumor tissues *vs* normal tissues and HCC serums *vs* normal serums. The meta-analysis of *p14*, *p15*, *p73*, *APC*, *SOCS1*, *MGMT*, *SFRP1*, *PRDM2*, *DAPK1*, *RARβ*, *IGF2* and *hMLH1* genes methylation was performed between HCC tumor tissues *vs* adjacent tissues and HCC tumor tissues *vs* normal tissues. The meta-analysis of *WIF1* gene methylation was performed between HCC tumor tissues vs adjacent tissues and HCC serums vs normal serums, and the meta-analysis of *DLC1*, *p53*, *SPINT2*, *RB1*, *OPCML* and *WT1* genes methylation was performed between HCC tumor tissues *vs* adjacent tissues.

**Figure 2:**
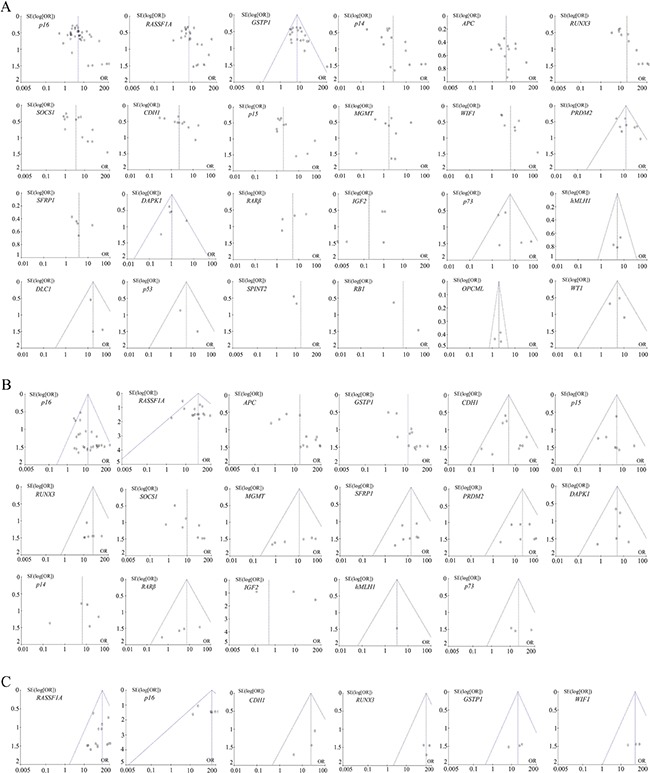
(**A**) Funnel plots of DNA methylation of 24 genes between HCC tumor tissues and adjacent tissues in the meta-analysis. (**B**) Funnel plots of DNA methylation of 17 genes between HCC tumor tissues and normal tissues in the meta-analysis. (**C**) Funnel plots of DNA methylation of four genes between HCC tumor serums and normal serums in the meta-analysis.

For 24 genes reported in at least three studies between HCC tumor tissues and adjacent tissues (Table 1), no evidence of statistical heterogeneity was observed for nine genes, including *GSTP1* (I^2^ = 35%), *PRDM2* (I^2^ = 40%), *DAPK1* (I^2^ = 19%), *p73* (I^2^ = 48%), *hMLH1* (I^2^ = 0%), *DLC1* (I^2^ = 0%), *p53* (I^2^ = 16%), *OPCML* (I^2^ = 0%) and *WT1* (I^2^ = 0%). No visual bias was shown in the meta-analysis of the above nine genes (Figure 2). Our data also demonstrated a significant heterogeneity of the remaining 15 genes that included *p16* (I^2^ = 68%), *RASSF1A* (I^2^ = 53%), *p14* (I^2^ = 77%), *APC* (I^2^ = 71%), *RUNX3* (I^2^ = 68%), *SOCS1* (I^2^ = 82%), *CDH1* (I^2^ = 82%), *p15* (I^2^ = 51%), *MGMT* (I^2^ = 79%), *WIF1* (I^2^ = 71%), *SFRP1* (I^2^ = 67%), *RARβ* (I^2^ = 62%), *IGF2* (I^2^ = 86%), *SPINT2* (I^2^ = 71%) and *RB1* (I^2^ = 64%). Therefore, random effect tests were applied for the meta-analysis of the above 15 genes. Their funnel plots were shown in Figure 2.

**Table 1:** Characteristics of 24 aberrant methylated genes between carcinoma tissues and adjacent tissues in HCC

Gene	Studies (n)	Overall OR (95% CI)	I^2^	*P* value	Carcinoma tissues/adjacent tissues
*p16*	43	5.10 [3.81, 6.84]	68%	< 0.00001	2185/2081
*RASSF1A*	28	6.70 [4.83, 9.30]	53%	< 0.00001	1414/1265
*GSTP1*	20	6.81 [5.39, 8.62]	35%	< 0.00001	1011/883
*p14*	17	2.67 [1.26, 5.64]	77%	0.01	911/717
*APC*	15	5.14 [3.18, 8.30]	71%	< 0.00001	888/762
*RUNX3*	13	19.99 [10.06, 39.72]	68%	< 0.00001	1025/998
*SOCS1*	13	3.47 [1.80, 6.71]	82%	0.0002	767/674
*CDH1*	12	2.31 [1.13, 4.74]	82%	0.02	575/533
*p15*	12	2.03 [1.23, 3.36]	51%	0.006	535/446
*MGMT*	10	1.66 [0.64, 4.28]	79%	0.3	560/470
*PRDM2*	9	12.33 [8.54, 17.81]	40%	< 0.00001	470/452
*WIF1*	8	6.53 [3.33, 12.80]	71%	< 0.00001	654/506
*DAPK1*	6	1.03 [0.64, 1.66]	19%	0.91	271/231
*SFRP1*	5	3.95 [1.91, 8.14]	67%	0.0002	304/275
*RARβ*	5	5.27 [1.53, 18.10]	62%	0.008	276/170
*IGF2*	5	0.18 [0.02, 1.55]	86%	0.12	160/200
*p73*	5	6.15 [3.09, 12.24]	48%	< 0.00001	275/169
*hMLH1*	4	5.10 [2.20, 11.85]	0%	0.0001	251/182
*DLC1*	3	17.30 [6.71, 44.58]	0%	< 0.00001	206/200
*p53*	3	5.12 [1.27, 20.59]	16%	0.02	107/107
*SPINT2*	3	18.38 [3.81, 88.61]	71%	0.0003	130/130
*RB1*	3	7.33 [0.58, 92.08]	64%	0.12	123/123
*OPCML*	3	1.93 [1.20, 3.11]	0%	0.006	213/144
*WT1*	3	5.08 [2.41, 10.69]	0%	< 0.0001	113/113

For 17 genes reported in at least three studies between HCC tumor tissues and normal tissues (Table 2), no evidence of statistical heterogeneity was observed for 11 genes, including *p16* (I^2^ = 13%), *RASSF1A* (I^2^ = 37%), *CDH1* (I^2^ = 17%), *p15* (I^2^ = 0%), *RUNX3* (I^2^ = 0%), *MGMT* (I^2^ = 36%), *SFRP1* (I^2^ = 0%), *PRDM2* (I^2^ = 0%), *DAPK1* (I^2^ = 0%), *RARβ* (I^2^ = 14%) and *p73* (I^2^ = 0%). No visual bias was shown in the meta-analysis of the above 11 genes (Figure 2). Our data also demonstrated a significant heterogeneity of 5 genes that included *APC* (I^2^ = 73%), *GSTP1* (I^2^ = 57%), *SOCS1* (I^2^ = 66%), *p14* (I^2^ = 56%) and *IGF2* (I^2^ = 89%). Therefore, random effect tests were applied for the meta-analysis of the above 5 genes. The heterogeneity of *hMLH1* was not applicable, because two of three case-control studies were not estimable. Their funnel plots were shown in Figure 2.

**Table 2:** Characteristics of 17 aberrant methylated genes between carcinoma tissues and normal tissues in HCC

Gene	Studies (n)	Overall OR (95% CI)	I^2^	*P* value	Carcinoma tissues/normal tissues
*p16*	31	13.41 [9.18, 19.59]	13%	< 0.00001	1415/399
*RASSF1A*	21	43.70 [26.92, 70.96]	37%	< 0.00001	900/341
*APC*	14	17.20 [5.75, 51.41]	73%	< 0.00001	727/217
*GSTP1*	13	13.56 [5.52, 33.29]	57%	< 0.00001	646/199
*CDH1*	10	4.93 [2.70, 8.99]	17%	< 0.00001	469/111
*p15*	9	5.48 [2.54, 11.79]	0%	< 0.0001	382/70
*RUNX3*	8	31.16 [12.24, 79.31]	0%	< 0.00001	549/152
*SOCS1*	8	9.73 [2.85, 33.27]	66%	0.0003	474/118
*MGMT*	8	10.84 [3.46, 33.91]	36%	< 0.0001	458/93
*PRDM2*	8	24.86 [10.44, 59.17]	0%	< 0.00001	422/116
*SFRP1*	7	13.97 [5.28, 36.92]	0%	< 0.00001	372/91
*DAPK1*	7	5.32 [2.38, 11.91]	0%	< 0.0001	328/77
*p14*	6	6.42 [1.54, 26.69]	56%	0.01	317/57
*RARβ*	5	7.37 [1.79, 30.39]	14%	0.006	230/54
*IGF2*	5	0.48 [0.02, 9.59]	89%	0.63	207/36
*hMLH1*	3	3.24 [0.18, 58.10]	—	0.42	204/34
*p73*	3	27.59 [4.87, 156.35]	0%	0.0002	174/27

The heterogeneity of *hMLH1* gene methylation was not applicable, because two of three case-control studies were not estimable.

For six genes reported in at least three studies between HCC tumor serums and normal serums (Table 3), no evidence of statistical heterogeneity was observed for four genes, including *RASSF1A* (I^2^ = 0%), *p16* (I^2^ = 0%), *CDH1* (I^2^ = 0%), *RUNX3* (I^2^ = 0%), *GSTP1* (I^2^ = 0%) and *WIF1* (I^2^ = 0%). No visual bias was shown in the meta-analysis of the above six genes and their funnel plots were shown in Figure 2.

**Table 3:** Characteristics of six aberrant methylated genes between carcinoma serums and normal serums in HCC

Gene	Studies (n)	Overall OR (95% CI)	I^2^	*P*value	Carcinoma serums/normal serums
*RASSF1A*	16	83.81 [47.35, 148.36]	0%	< 0.00001	1110/783
*p16*	9	123.15 [49.12, 308.74]	0%	< 0.00001	510/352
*CDH1*	3	23.70 [5.39, 104.28]	0%	< 0.0001	216/90
*RUNX3*	3	103.92 [16.33, 661.45]	0%	< 0.00001	172/110
*GSTP1*	3	21.09 [4.02, 110.65]	0%	0.0003	155/100
*WIF1*	3	53.65 [10.62, 271.09]	0%	< 0.00001	252/180

As shown in Table 1, the meta-analysis of *p16* gene was involved with 43 studies between 2185 HCC tumor tissues and 2081 adjacent tissues. Our results revealed that the frequency of *p16* gene methylation in carcinoma tissues was significantly higher than adjacent tissues (the overall OR = 5.10, 95% CI = 3.81–6.84, *p* < 0.00001). The meta-analysis of *RASSF1A* methylation between 1414 HCC tumor tissues and 1265 adjacent tissues indicated a statistical difference (the overall OR = 6.70, 95% CI = 4.83–9.30, *p* < 0.00001). The same consequence was also found in the other 18 genes including *APC* (the overall OR = 5.14, 95% CI = 3.18–8.30, *p* < 0.00001), *RUNX3* (the overall OR = 19.99, 95% CI = 10.06–39.72, *p* < 0.00001), *SOCS1* (the overall OR = 3.47, 95% CI = 1.80–6.71, *p* = 0.0002), *CDH1* (the overall OR = 2.31, 95% CI = 1.13–4.74, *p* = 0.02), *p15* (the overall OR = 2.03, 95% CI = 1.23–3.36, p = 0.006), *WIF1* (the overall OR = 6.53, 95% CI = 3.33–12.80, *p* < 0.00001), *PRDM2* (the overall OR = 12.33, 95% CI = 8.54–17.81, *p* < 0.00001), *SFRP1* (the overall OR = 3.95, 95% CI = 1.91–8.14, *p* = 0.0002), *RARβ* (the overall OR = 5.27, 95% CI = 1.53–18.10, *p* = 0.008), *p73* (the overall OR = 6.15, 95% CI = 3.09–12.24, *p* < 0.00001), *hMLH1* (the overall OR = 5.10, 95% CI = 2.20–11.85, *p* = 0.0001), *DLC1* (the overall OR = 17.30, 95% CI = 6.71–44.58, *p* < 0.00001), *p53* (the overall OR = 5.12, 95% CI = 1.27–20.59, *p* = 0.02), *SPINT2* (the overall OR = 18.38, 95% CI = 3.81–88.61, *p* = 0.0003), *OPCML* (the overall OR = 1.93, 95% CI = 1.20–3.11, *p* = 0.006) and *WT1* (the overall OR = 5.08, 95% CI = 2.41–10.69, *p* < 0.0001). Our meta-analysis was unable to find any statistical significance between HCC tumor tissues and adjacent tissues for the methylation of the remaining four genes, including *MGMT*, *DAPK1*, *IGF2* and *RB1*.

As shown in Table 2, the meta-analysis of *p16* gene was involved with 31 studies between 1415 HCC tumor tissues and 399 normal tissues. Our results revealed that the frequency of *p16* gene methylation in carcinoma tissues was significantly higher than normal tissues (the overall OR = 13.41, 95% CI = 9.18–19.59, *p* < 0.00001). The meta-analysis of *RASSF1A* methylation between 900 HCC tumor tissues and 341 normal tissues indicated a statistical difference (the overall OR = 43.70, 95% CI = 26.92–70.96, *p* < 0.00001). The same consequence was also found in the other 13 genes including *APC* (the overall OR = 17.20, 95% CI = 5.75–51.41, *p* < 0.00001), *GSTP1* (the overall OR = 13.56, 95% CI = 5.52–33.29, *p* < 0.00001), *CDH1* (the overall OR = 4.93, 95% CI = 2.70–8.99, *p* < 0.00001), *p15* (the overall OR = 5.48, 95% CI = 2.54–11.79, *p* < 0.0001), *RUNX3* (the overall OR = 31.16, 95% CI = 12.24–79.31, *p* < 0.00001), *SOCS1* (the overall OR = 9.73, 95% CI = 2.85–33.27, *p* = 0.0003), *MGMT* (the overall OR = 10.84, 95% CI = 3.46–33.91, *p* < 0.0001), *SFRP1* (the overall OR = 13.97, 95% CI = 5.28–36.92, *p* < 0.00001), *PRDM2* (the overall OR = 24.86, 95% CI = 10.44–59.17, *p* < 0.00001), *DAPK1* (the overall OR = 5.32, 95% CI = 2.38–11.91, *p* < 0.0001), *p14* (the overall OR = 6.42, 95% CI = 1.54–26.69, *p* = 0.01), *RARβ* (the overall OR = 7.37, 95% CI = 1.79–30.39, *p* = 0.006) and *p73* (the overall OR = 27.59, 95% CI = 4.87–156.35, *p* = 0.0002). Our meta-analysis was unable to find any statistical significance between HCC tumor tissues and adjacent tissues for the methylation of the remaining two genes, including *IGF2* and *hMLH1*.

As shown in Table 3, Our meta-analysis showed statistical significance between HCC tumor serums and normal serums for the methylation of all six genes, including *RASSF1A* (the overall OR = 83.81, 95% CI = 47.35–148.36, *p* < 0.00001), *p16* (the overall OR = 123.15, 95% CI = 49.12–308.74, *p* < 0.00001), *CDH1* (the overall OR = 23.70, 95% CI = 5.39–104.28, *p* < 0.0001), *RUNX3* (the overall OR = 103.92, 95% CI = 16.33–661.45, *p* < 0.00001), *GSTP1* (the overall OR = 21.09, 95% CI = 4.02–110.65, *p* = 0.0003) and *WIF1* (the overall OR = 53.65, 95% CI = 10.62–271.09, *p* < 0.00001).

In this meta-analysis, we selected geographical populations to analyze sources of heterogeneity. We found that there was significant difference in *RASSF1A* and *GSTP1* between HCC tumor tissues and normal tissues in Japan (*p* = 0.041) and America (*p* = 0.021) respectively (Supplementary Table S2). However, there was no significance in other genes between HCC tumor tissues and normal tissues in geographical populations (Supplementary Table S2). In addition, we didn't find any significance in 24 methylated genes between HCC tumor tissues and adjacent tissues (Supplementary Table S1) and six methylated genes between HCC tumor serums and normal serums (Supplementary Table S3) in geographical populations. Our meta-analysis indicated that the heterogeneity was not contributed by geographical populations, except *RASSF1A* and *GSTP1* between HCC tumor tissues and normal tissues in Japan and America respectively.

**Figure 3:**
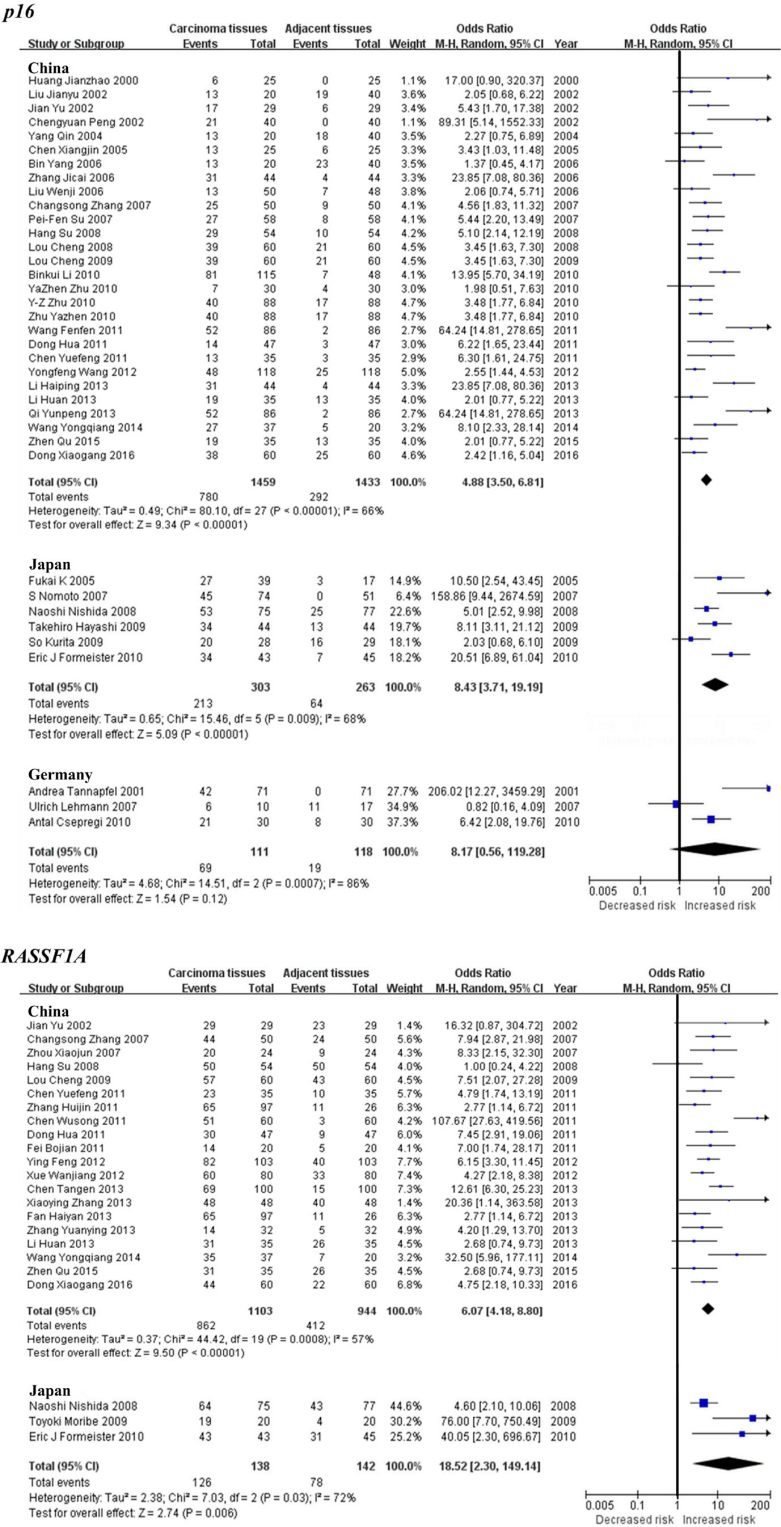
Forest plots of *p16* and *RASSF1A* methylation between HCC tumor tissues and adjacent tissues in the meta-analysis

**Figure 4:**
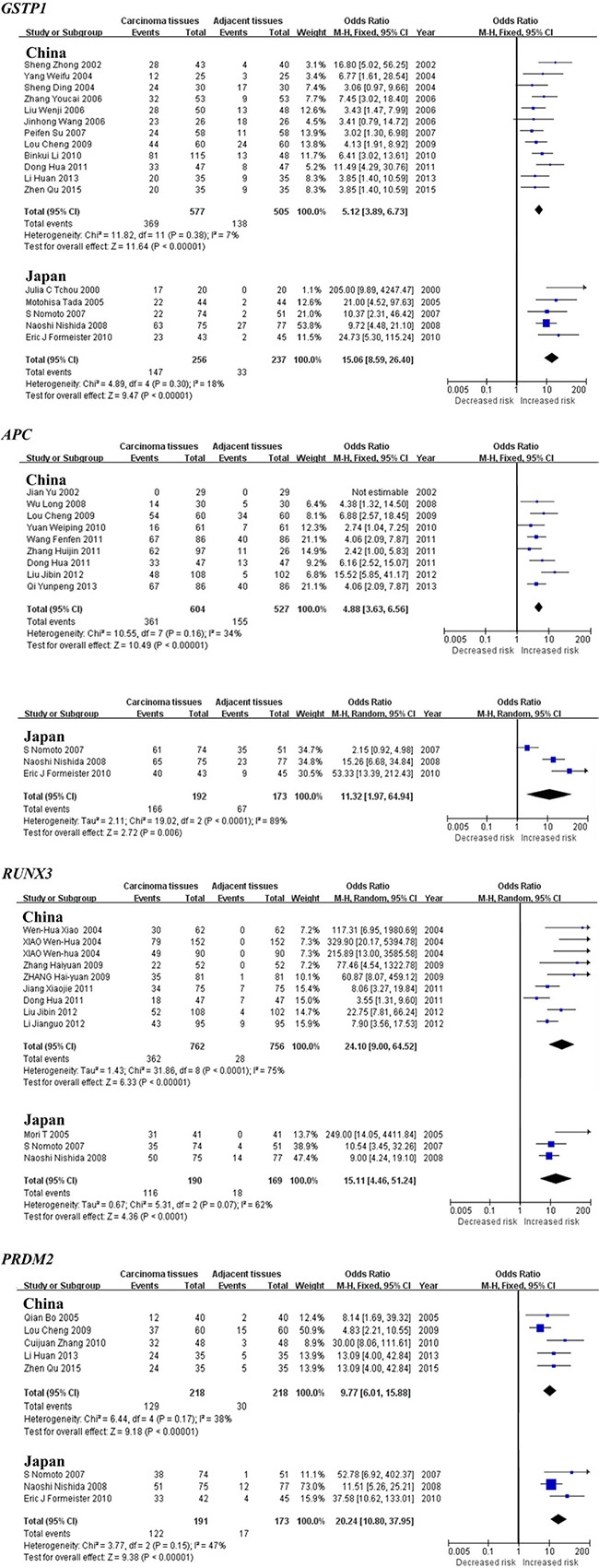
Forest plots of *GSTP1*, *APC*, *RUNX3* and *PRDM2* methylation between HCC tumor tissues and adjacent tissues in the meta-analysis

Subgroup meta-analysis by geographical populations was performed for *p16*, *RASSF1A*, *GSTP1*, *APC*, *RUNX3*, *SOCS1* and *PRDM2* between HCC tumor tissues and adjacent tissues. As shown in Figure 3, we found a statistical difference between HCC tumor tissues and adjacent tissues for *p16* methylation in China from 28 studies (OR = 4.88, 95% CI = 3.50–6.81, I^2^ = 66%, *p* < 0.00001) and in Japan from 6 studies (OR = 8.43, 95% CI = 3.71–19.19, I^2^ = 68%, *p* < 0.00001), but not in Germany from 3 studies (*p* = 0.12, I^2^ = 86%). In addition, there was a significant geographical difference in the meta-analysis of *RASSF1A* gene in China (OR = 6.07, 95% CI = 4.18–8.80, I^2^ = 57%, *p* < 0.00001) and Japan (OR = 18.52, 95% CI = 2.30–149.14, I^2^ = 72%, *p* = 0.006, Figure 3). The same consequence was also found in the other four genes including *GSTP1* (China: OR = 5.12, 95% CI = 3.89–6.73, I^2^ = 7%, *p* < 0.00001; Japan: OR = 15.06, 95% CI = 8.59–26.40, I^2^ = 18%, *p* < 0.00001), *APC* (China: OR = 4.88, 95% CI = 3.63–6.56, I^2^ = 34%, *p* < 0.00001; Japan: OR = 11.32, 95% CI = 1.97–64.94, I^2^ = 89%, *p* = 0.006), *RUNX3* (China: OR = 24.10, 95% CI = 9.00–64.52, I^2^ = 75%, *p* < 0.00001; Japan: OR = 15.11, 95% CI = 4.46–51.24, I^2^ = 62%, *p* < 0.0001) and *PRDM2* (China: OR = 9.77, 95% CI = 6.01–15.88, I^2^ = 38%, *p* < 0.00001; Japan: OR = 20.24, 95% CI = 10.80–37.95, I^2^ = 47%, *p* < 0.00001)(Figure 4). The subgroup meta-analysis of *SOCS1* was unable to observe any significant result in each geographical population (Supplementary Figure S1).

**Figure 5:**
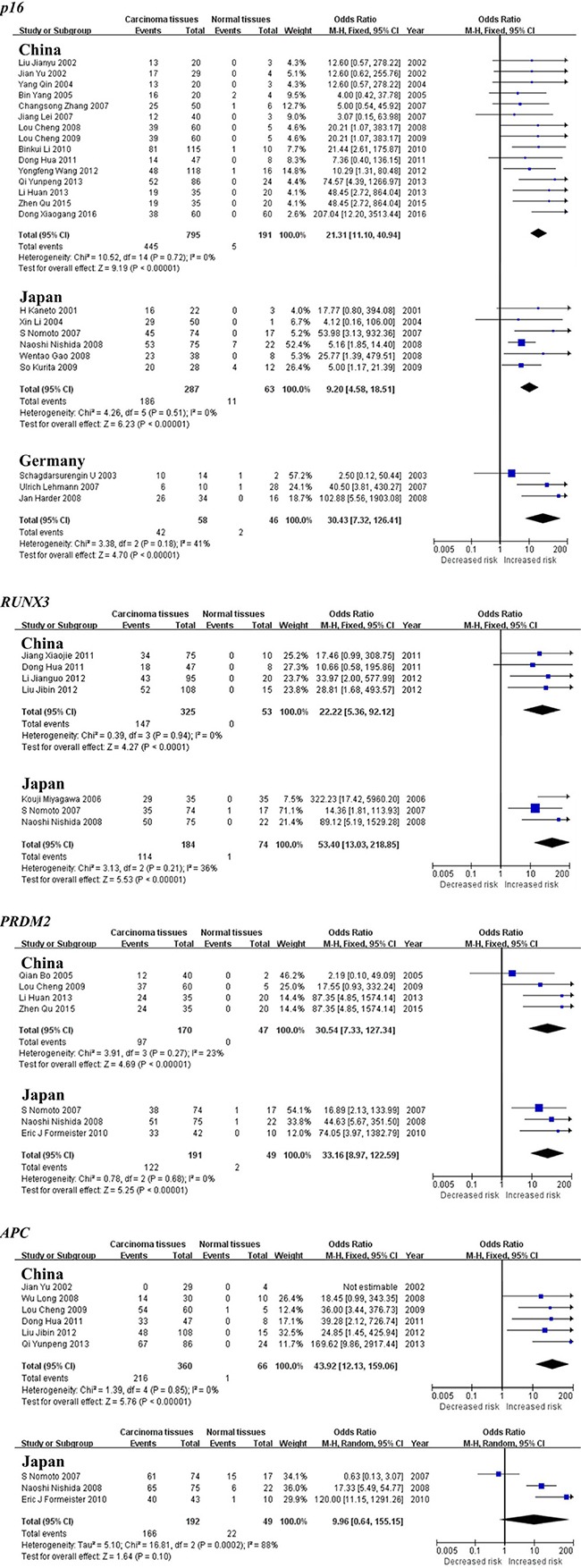
Forest plots of *p16*, *RUNX3*, *PRDM2* and *APC* methylation between HCC tumor tissues and normal tissues in the meta-analysis

The subgroup meta-analysis by geographical populations was also performed for *p16*, *APC*, *RUNX3* and *PRDM2* between HCC tumor tissues and normal tissues. As shown in Figure 5, we found a statistical difference between HCC tumor tissues and normal tissues for *p16* methylation in China from 15 studies (OR = 21.31, 95% CI = 11.10–40.94, I^2^ = 0%, *p* < 0.00001), in Japan from 6 studies (OR = 9.20, 95% CI = 4.58–18.51, I^2^ = 0%, *p* < 0.00001) and in Germany from 3 studies (OR = 30.43, 95% CI = 7.32–126.41, I^2^ = 41%, *p* < 0.00001). The same consequence was also found in the other two genes including *RUNX3* (China: OR = 22.22, 95% CI = 5.36–92.12, I^2^ = 0%, *p* < 0.0001; Japan: OR = 53.40, 95% CI = 13.03–218.85, I^2^ = 36%, *p* < 0.00001) and *PRDM2* (China: OR = 30.54, 95% CI = 7.33–127.34, I^2^ = 23%, *p* < 0.00001; Japan: OR = 33.16, 95% CI = 8.97–122.59, I^2^ = 0%, *p* < 0.00001)(Figure 5). In addition, there was a significant geographical difference in the meta-analysis of *APC* gene in China (OR = 43.92, 95% CI = 12.13–159.06, I^2^ = 0%, *p* < 0.00001) but not in Japan (OR = 9.96, 95% CI = 0.64–155.15, I^2^ = 88%, *p* = 0.10, Figure 5).

## DISCUSSION

Our meta-analysis has included a large amount of studies that evaluated the contribution of aberrant DNA methylation to the risk of HCC. The meta-analysis mainly focused on 24 tumor suppressor genes between HCC tumor tissues and adjacent tissues, 17 tumor suppressor genes between HCC tumor tissues and normal tissues and six tumor suppressor genes between HCC serums and normal serums (≥3 studies per gene). 20 HCC-associated genes (*p16*, *RASSF1A*, *GSTP1*, *p14*, *APC*, *RUNX3*, *SOCS1*, *CDH1*, *p15*, *WIF1*, *PRDM2*, *SFRP1*, *RARβ*, *p73*, *hMLH1*, *DLC1*, *p53*, *SPINT2*, *OPCML* and *WT1*) hypermethylation showed significant evidences between HCC tumor tissues and adjacent tissues to the risk of HCC. 15 HCC-associated genes (*p16*, *RASSF1A*, *APC*, *GSTP1*, *CDH1*, *p15*, *RUNX3*, *SOCS1*, *MGMT*, *SFRP1*, *PRDM2*, *DAPK1*, *p14*, *RARβ* and *p73*) hypermethylation showed significant evidences between HCC tumor tissues and normal tissues to the risk of HCC. Six HCC-associated genes (*RASSF1A*, *p16*, *CDH1*, *RUNX3*, *GSTP1* and *WIF1*) hypermethylation showed significant evidences between HCC serums and normal serums to the risk of HCC.

This meta-analysis showed that methylation of *p16*, *RASSF1A*, *APC*, *GSTP1*, *CDH1*, *p15*, *RUNX3*, *SOCS1*, *SFRP1*, *PRDM2*, *p14*, *RARβ* and *p73* genes in HCC tumor tissues was significantly higher than both adjacent tissues and normal tissues, revealing that the number of hepatic cells with these methylated genes may increase significantly in hepatocarcinogenesis process from normal liver to adjacent liver and HCC. In addition, methylation of *MGMT* and *DAPK1* genes in HCC tumor tissues was significantly higher than normal tissues but not adjacent tissues, revealing that methylation of two genes may play a significant role in the early stage of hepatocarcinogenesis process. However, methylation of *IGF2* gene in HCC tumor tissues was not significantly higher than neither adjacent tissues nor normal tissues, revealing that methylation of *IGF2* gene didn't play a significant role during the hepatocarcinogenesis process.

Subgroup meta-analysis by geographical populations found that *RASSF1A*, *GSTP1*, *APC*, *RUNX3* and *PRDM2* hypermethylation was a common risk factor between HCC tumor tissues and adjacent tissues in HCC patients, however, the statistical significance of *p16* hypermethylation was only found in China and Japan, but not in Germany. In addition, we found that subgroup meta-analysis by geographical populations showed *p16*, *RUNX3* and *PRDM2* hypermethylation was a common risk factor between HCC tumor tissues and normal tissues in HCC patients. However, the statistical significance of *APC* hypermethylation was only found in China but not in Japan. The effects of *p16* hypermethylation on China, Japan and Germany, and *APC* hypermethylation on China and Japan are different, due to the diversities of hereditary, alcohol consumption and hepatitis virus infection in the different geographic regions [4, 19, 20]. Our results suggested that aberrant DNA methylation might become useful biomarkers in the early diagnosis of HCC.

On the other hand, some meta-analyses showed that global DNA hypomethylation in peripheral blood leukocytes may be a promising biomarker for cancer risk [21]. The association between global DNA methylation and cancer risk may depend on different factors, such as cancer and sample types [22]. For example, Barchitta found that LINE-1 methylation, a representative biomarker for global DNA methylation, increased significantly in colorectal carcinoma and gastric carcinoma, but not hepatocellular carcinoma. In addition, the significant difference in methylation levels was confirmed in tissue samples, but not in blood samples [22]. However, our meta-analysis showed that methylation of four genes (*RASSF1A*, *p16*, *CDH1* and *RUNX3*) in both tissue and serum samples were significantly different for the risk of HCC.

Aberrant gene methylation is recognized as one of the main mechanisms of triggering HCC [23], and it may serve as a useful biomarker for the prediction of HCC risk. The silencing of tumor suppressor genes by hypermethylation in the promoter regions also contributed to HCC progression [24]. For example, *RASSF1A* promoter methylation may conduce to the loss of RASSF1A expression, thereby leading to the silencing of *RASSF1A* gene and decrease of its function, which is one of the most common early events in HCC [25]. The same phenomenon occurred in many other genes, such as *p16*, *GSTP1*, *APC*, *RUNX3*, *SOCS1*, *WIF1*, *p73*, *hMLH1*, *DLC1*, *OPCML* and *WT1* [18, 26–33]. The frequencies of methylation of these gene promoters were significant higher in HCC than in nonneoplastic liver tissues and normal liver tissues. In addition, Feng revealed that *HOXA9*, *RASSF1* and *SFRP1* were more frequently methylated in HBV-positive HCC cases, while *CDKN2A* were significantly more frequently methylated in HCV-positive HCC cases, suggesting that methylation of these gene promoters may be involved in virus-induced hepatocarcinogenesis [34].

The present meta-analysis has several limitations that need to be taken with the following cautions. First, the selection bias is inevitable due to the search strategy restricted to the articles published in English or Chinese. Secondly, the heterogeneity existed in 15 genes between HCC tumor tissues and adjacent tissues and five genes between HCC tumor tissues and normal tissues. This phenomenon may be caused by the moderate number of samples in the involved studies and the inconsistent criteria in the selection of controls. We expect a larger size of samples to be tested in the future for a more reliable conclusion. Thirdly, our meta-analysis focused on the genes with at least three independent studies, and this might prevent those genes reported in two large studies from being included in the current meta-analysis. Finally, the status of DNA methylation was qualitative (M+ or M−) in all the selected studies that were performed with methylation specific PCR (MSP). The tested CpG sites might not stand for the whole promoter regions.

In summary, our meta-analysis indicated that aberrant DNA methylation was associated with the risk of HCC. Aberrant DNA methylation might become useful biomarkers for the prediction and prognostication of HCC.

## MATERIALS AND METHODS

### Study identification

We conducted a comprehensive systematic literature via PubMed, Web of Science, Cochrane Library, Embase, CNKI and Wanfang using the “hepatocellular carcinoma or hepatocarcinoma or primary liver cancer or HCC or hepatic carcinoma or liver tumor” and “DNA methylation” as keywords in titles and abstracts. The search was updated until September 1, 2016. The search was limited to the articles published in English and Chinese. A preliminary review of abstracts was conducted to determine the relevance on methylation study. Studies were selected if they met the following criteria: (1) they were the case-control associations of gene methylation with the risk of HCC in humans; (2) they had the sufficient methylation informations to calculate the odds ratios (ORs) and 95 % confidence intervals (CIs) for the meta-analysis. We excluded the studies such as letters, reviews, abstracts and conference articles. Studies with non case-control or no methylation frequency data were also removed from the meta-analysis. Furthermore, we excluded the genes with the number of articles less than 3. The selection process of the included studies was shown in the flow chart of Figure 1.

### Data extraction and quality assessment

All the literatures and data included in the meta-analysis were retrieved and extracted independently by five authors (CZ, JL, TH, DD and DJ) to improve subjective bias, and any discrepancy was checked again and resolved through discussion. For each eligible article, we extracted the following information: first author's name, publication year, geographical populations, the numbers of cases and controls.

Quality assessment was performed by three authors (CZ, XS and DL) independently and any disagreement was discussed with the fourth author (KW). Due to the observational study design of the included studies, the Newcastle-Ottawa Scale was used to assess the methodological quality of the included studies. It assessed the selection, comparability and exposure of a case-control study.

### Meta-analysis

The Review Manager software (version 5.0, Cochrane Collaboration, Oxford, UK) and STATA (version 10.0 Stata Corporation College Station, TX) were used for the current meta-analysis. The combined ORs and the corresponding 95% CIs were calculated and computed in the forest plots for each gene to evaluate the contribution of its DNA methylation to the risk of HCC. A fixed-effect model was applied for the meta-analysis with the moderate heterogeneity (I^2^ < 50%), otherwise a random-effect model was used. Funnel plots were used to check whether there was an obvious publication bias among the involved studies. *P* values less than 0.05 were considered to be significant.

## SUPPLEMENTARY MATERIALS







## Abbreviations

HCC: Hepatocellular carcinoma
CNKI: China National Knowledge Infrastructure
OR: Odds ratio
CI: Confidence interval
RASSF1A: Ras association domain family member1A
GSTP1: Glutathione S-transferase pi 1
APC: Adenomatous polyposis coli
RUNX3: Runt-related transcription factor 3
SOCS1: Suppressor of the cytokine signalling 1
MGMT: O6-methylguanine-DNA methyltransferase
WIF1: WNT inhibitory factor 1
PRDM2: PR domain containing 2
SFRP1: Secreted frizzled-related protein 1
RARβ: Retinoic acid receptor beta
hMLH1: Human MutL homolog 1
DLC1: Deleted in liver cancer 1
SPINT2: Serine peptidase inhibitor, Kunitz type, 2
OPCML: Opioid binding protein/cell adhesion molecule-like
WT1: Wilms tumor 1
