# Supplementary material for: Meta-analysis of DNA methylation biomarkers in hepatocellular carcinoma
Source: Oncotarget. 2016 Nov 8;7(49):81255–67. doi: 10.18632/oncotarget.13221 (PMC5348390; doi:10.18632/oncotarget.13221)
Supplement: Supplementary file 1 [file oncotarget-07-81255-s001.pdf]

Meta-analysis of DNA methylation biomarkers in hepatocellular carcinoma

Supplementary Materials

Supplementary Document S1: 144 eligible studies in the meta-analysis

REFERENCES

1. Tannapfel A, Busse C, Weinans L, Benicke M, Katalinic A, Geissler F, Hauss J, Wittekind C. INK4a-ARF alterations and p53 mutations in hepatocellular carcinomas. Oncogene. 2001; 20:7104–9.
2. Peng CY, Chen TC, Hung SP, Chen MF, Yeh CT, Tsai SL, Chu CM, Liaw YF. Genetic alterations of INK4alpha/ARF locus and p53 in human hepatocellular carcinoma. Anticancer Res. 2002; 22:1265–71.
3. Yu J, Ni M, Xu J, Zhang H, Gao B, Gu J, Chen J, Zhang L, Wu M, Zhen S, Zhu J. Methylation profiling of twenty promoter-CpG islands of genes which may contribute to hepatocellular carcinogenesis. BMC Cancer. 2002; 2:29.
4. Yang B, Guo M, Herman JG, Clark DP. Aberrant promoter methylation profiles of tumor suppressor genes in hepatocellular carcinoma. Am J Pathol. 2003; 163:1101–7.
5. Fukai K, Yokosuka O, Imazeki F, Tada M, Mikata R, Miyazaki M, Ochiai T, Saisho H: Methylation status of p14ARF, p15INK4b, and p16INK4a genes in human hepatocellular carcinoma. Liver Int. 2005; 25:1209–16.
6. Liu WJ, Wang L, Wang JP, Li JQ, Zhang CQ, Zheng L, Yuan YF. Correlations of CpG island methylator phenotype and OPCML gene methylation to carcinogenesis of hepatocellular carcinoma. Ai Zheng. 2006; 25:696–700.
7. Zhang C, Li Z, Cheng Y, Jia F, Li R, Wu M, Li K, Wei L. CpG island methylator phenotype association with elevated serum alpha-fetoprotein level in hepatocellular carcinoma. Clin Cancer Res. 2007; 13:944–52.
8. Su PF, Lee TC, Lin PJ, Lee PH, Jeng YM, Chen CH, Liang JD, Chiou LL, Huang GT, Lee HS. Differential DNA methylation associated with hepatitis B virus infection in hepatocellular carcinoma. Int J Cancer. 2007; 121:1257–64.
9. Vivekanandan P, Torbenson M. Epigenetic instability is rare in fibrolamellar carcinomas but common in viral-associated hepatocellular carcinomas. Mod Pathol. 2008; 21:670–5.
10. Hayashi T, Tamori A, Nishikawa M, Morikawa H, Enomoto M, Sakaguchi H, Habu D, Kawada N, Kubo S, Nishiguchi S, Shiomi S. Differences in molecular alterations of hepatocellular carcinoma between patients with a sustained virological response and those with hepatitis C virus infection. Liver Int. 2009; 29:126–32.
11. Herath NI, Purdie DM, Kew MC, Smith JL, Young J, Leggett BA, MacDonald GA. Varying etiologies lead to different molecular changes in Australian and South African hepatocellular carcinomas. Int J Oncol. 2009; 35:1081–9.
12. Li B, Liu W, Wang L, Li M, Wang J, Huang L, Huang P, Yuan Y. CpG island methylator phenotype associated with tumor recurrence in tumor-node-metastasis stage I hepatocellular carcinoma. Ann Surg Oncol. 2010; 17:1917–26.
13. Zekri AR, Bahnasy AA, Shoeab FE, Mohamed WS, El-Dahshan DH, Ali FT, Sabry GM, Dasgupta N, Daoud SS. Methylation of multiple genes in hepatitis C virus associated hepatocellular carcinoma. J Adv Res. 2014; 5:27–40.
14. Kaneto H, Sasaki S, Yamamoto H, Itoh F, Toyota M, Suzuki H, Ozeki I, Iwata N, Ohmura T, Satoh T, Karino Y, Satoh T, Toyota J, et al. Detection of hypermethylation of the p16(INK4A) gene promoter in chronic hepatitis and cirrhosis associated with hepatitis B or C virus. Gut. 2001; 48:372–7.
15. Schagdarsurengin U, Wilkens L, Steinemann D, Flemming P, Kreipe HH, Pfeifer GP, Schlegelberger B, Dammann R. Frequent epigenetic inactivation of the RASSF1A gene in hepatocellular carcinoma. Oncogene. 2003; 22:1866–71.
16. Lee S, Lee HJ, Kim JH, Lee HS, Jang JJ, Kang GH: Aberrant CpG island hypermethylation along multistep hepatocarcinogenesis. Am J Pathol. 2003; 163:1371–8.
17. Anzola M, Cuevas N, Lopez-Martinez M, Martinez de Pancorbo M, Burgos JJ. p16INK4A gene alterations are not a prognostic indicator for survival in patients with hepatocellular carcinoma undergoing curative hepatectomy. J Gastroenterol Hepatol. 2004; 19:397–405.
18. Qin Y, Liu JY, Li B, Sun ZL, Sun ZF. Association of low p16INK4a and p15INK4b mRNAs expression with their CpG islands methylation with human hepatocellular carcinogenesis. World J Gastroenterol. 2004; 10:1276–80.

19. Li X, Hui AM, Sun L, Hasegawa K, Torzilli G, Minagawa M, Takayama T, Makuuchi M. p16INK4A hypermethylation is associated with hepatitis virus infection, age, and gender in hepatocellular carcinoma. Clin Cancer Res. 2004; 10:7484–9.
20. Park HJ, Yu E, Shim YH. DNA methyltransferase expression and DNA hypermethylation in human hepatocellular carcinoma. Cancer Lett. 2006; 233:271–8.
21. Yang B, Gao YT, Du Z, Zhao L, Song WQ. Methylation-based molecular margin analysis in hepatocellular carcinoma. Biochem Biophys Res Commun. 2005; 338:1353–8.
22. Katoh H, Shibata T, Kokubu A, Ojima H, Fukayama M, Kanai Y, Hirohashi S. Epigenetic instability and chromosomal instability in hepatocellular carcinoma. Am J Pathol. 2006; 168:1375–84.
23. Jicai Z, Zongtao Y, Jun L, Haiping L, Jianmin W, Lihua H. Persistent infection of hepatitis B virus is involved in high rate of p16 methylation in hepatocellular carcinoma. Mol Carcinog. 2006; 45:530–6.
24. Lehmann U, Wingen LU, Brakensiek K, Wedemeyer H, Becker T, Heim A, Metzig K, Hasemeier B, Kreipe H, Flemming P. Epigenetic defects of hepatocellular carcinoma are already found in non-neoplastic liver cells from patients with hereditary haemochromatosis. Hum Mol Genet. 2007; 16:1335–42.
25. Zhang YJ, Wu HC, Shen J, Ahsan H, Tsai WY, Yang HI, Wang LY, Chen SY, Chen CJ, Santella RM. Predicting hepatocellular carcinoma by detection of aberrant promoter methylation in serum DNA. Clin Cancer Res. 2007; 13:2378–84.
26. Oh BK, Kim H, Park HJ, Shim YH, Choi J, Park C, Park YN. DNA methyltransferase expression and DNA methylation in human hepatocellular carcinoma and their clinicopathological correlation. Int J Mol Med. 2007; 20:65–73.
27. Dejeux E, Audard V, Cavard C, Gut IG, Terris B, Tost J. Rapid identification of promoter hypermethylation in hepatocellular carcinoma by pyrosequencing of etiologically homogeneous sample pools. J Mol Diagn. 2007; 9:510–20.
28. Tan SH, Ida H, Lau QC, Goh BC, Chieng WS, Loh M, Ito Y. Detection of promoter hypermethylation in serum samples of cancer patients by methylation-specific polymerase chain reaction for tumour suppressor genes including RUNX3. Oncol Rep. 2007; 18:1225–30.
29. Nomoto S, Kinoshita T, Kato K, Otani S, Kasuya H, Takeda S, Kanazumi N, Sugimoto H, Nakao A. Hypermethylation of multiple genes as clonal markers in multicentric hepatocellular carcinoma. Br J Cancer. 2007; 97:1260–5.
30. Nishida N, Nagasaka T, Nishimura T, Ikai I, Boland CR, Goel A. Aberrant methylation of multiple tumor suppressor genes in aging liver, chronic hepatitis, and hepatocellular carcinoma. Hepatology. 2008; 47:908–18.
31. Harder J, Opitz OG, Brabender J, Olschewski M, Blum HE, Nomoto S, Usadel H. Quantitative promoter methylation analysis of hepatocellular carcinoma, cirrhotic and normal liver. Int J Cancer. 2008; 122:2800–4.
32. Su H, Zhao J, Xiong Y, Xu T, Zhou F, Yuan Y, Zhang Y, Zhuang SM: Large-scale analysis of the genetic and epigenetic alterations in hepatocellular carcinoma from Southeast China. Mutat Res. 2008; 641:27–35.
33. Gao W, Kondo Y, Shen L, Shimizu Y, Sano T, Yamao K, Natsume A, Goto Y, Ito M, Murakami H, Osada H, Zhang J, Issa JP, et al. Variable DNA methylation patterns associated with progression of disease in hepatocellular carcinomas. Carcinogenesis. 2008; 29:1901–10.
34. Kurita S, Ohkoshi S, Yano M, Yamazaki K, Suzuki K, Aoki YH, Matsuda Y, Wakai T, Shirai Y, Ichida T, Aoyagi Y. Progression of hypermethylation of the p16(INK4A) gene from normal liver to nontumorous liver and hepatocellular carcinoma: an evaluation using quantitative PCR analysis. Dig Dis Sci. 2009; 54:80–8.
35. Lou C, Du Z, Yang B, Gao Y, Wang Y, Fang S: Aberrant DNA methylation profile of hepatocellular carcinoma and surgically resected margin. Cancer Sci. 2009; 100:996–1004.
36. Zhu YZ, Zhu R, Fan J, Pan Q, Li H, Chen Q, Zhu HG. Hepatitis B virus X protein induces hypermethylation of p16(INK4A) promoter via DNA methyltransferases in the early stage of HBV-associated hepatocarcinogenesis. J Viral Hepat. 2010; 17:98–107.
37. Feng Q, Stern JE, Hawes SE, Lu H, Jiang M, Kiviat NB. DNA methylation changes in normal liver tissues and hepatocellular carcinoma with different viral infection. Exp Mol Pathol. 2010; 88:287–92.
38. Csepregi A, Ebert MP, Röcken C, Schneider-Stock R, Hoffmann J, Schulz HU, Roessner A, Malfertheiner P. Promoter methylation of CDKN2A and lack of p16 expression characterize patients with hepatocellular carcinoma. BMC Cancer. 2010; 10:317.
39. Zhu YZ, Zhu R, Shi LG, Mao Y, Zheng GJ, Chen Q, Zhu HG. Hepatitis B virus X protein promotes hypermethylation of p16(INK4A) promoter through upregulation of DNA methyltransferases in hepatocarcinogenesis. Exp Mol Pathol. 2010; 89.268–75.
40. Formeister EJ, Tsuchiya M, Fujii H, Shpyleva S, Pogribny IP, Rusyn I. Comparative analysis of promoter methylation and gene expression endpoints between tumorous and non-tumorous tissues from HCV-positive patients with hepatocellular carcinoma. Mutat Res. 2010; 692:26–33.
41. Hua D, Hu Y, Wu YY, Cheng ZH, Yu J, Du X, Huang ZH. Quantitative methylation analysis of multiple genes using methylation-sensitive restriction enzyme-based quantitative PCR for the detection of hepatocellular carcinoma. Exp Mol Pathol. 2011; 91:455–60.
42. Wang Y, Cheng J, Xu C, Liu S, Jiang S, Xu Q, Chen X, Zhuang H, Lu F. Quantitative methylation analysis reveals gender and age differences in p16INK4a hypermethylation in hepatitis B virus-related hepatocellular carcinoma. Liver Int. 2012; 32:420–8.

43. Qu Z, Jiang Y, Li H, Yu DC, Ding YT. Detecting abnormal methylation of tumor suppressor genes GSTP1, P16, RIZ1, and RASSF1A in hepatocellular carcinoma and its clinical significance. Oncol Lett. 2015; 10:2553–2558.

44. Yeo W, Wong N, Wong WL, Lai PB, Zhong S, Johnson PJ. High frequency of promoter hypermethylation of RASSF1A in tumor and plasma of patients with hepatocellular carcinoma. Liver Int. 2005; 25:266–72.

45. Calvisi DF, Ladu S, Gorden A, Farina M, Conner EA, Lee JS, Factor VM, Thorgeirsson SS. Ubiquitous activation of Ras and Jak/Stat pathways in human HCC. Gastroenterology. 2006; 130:1117–28.

46. Chan KC, Lai PB, Mok TS, Chan HL, Ding C, Yeung SW, Lo YM. Quantitative analysis of circulating methylated DNA as a biomarker for hepatocellular carcinoma. Clin Chem. 2008; 54:1528–36.

47. Saelee P, Wongkham S, Chariyalertsak S, Petmitr S, Chuensumran U. RASSF1A promoter hypermethylation as a prognostic marker for hepatocellular carcinoma. Asian Pac J Cancer Prev. 2010; 11:1677–81.

48. Feng Y, Xue WJ, Li P, Sha ZY, Huang H, Rui L, Li HX, Mao QS. RASSF1A hypermethylation is associated with aflatoxin B1 and polycyclic aromatic hydrocarbon exposure in hepatocellular carcinoma. Hepatogastroenterology. 2012; 59:1883–8.

49. Zhang X, Li HM, Liu Z, Zhou G, Zhang Q, Zhang T, Zhang J, Zhang C. Loss of heterozygosity and methylation of multiple tumor suppressor genes on chromosome 3 in hepatocellular carcinoma. J Gastroenterol. 2013; 48:132–43.

50. Mohamed NA, Swify EM, Amin NF, Soliman MM, Tag-Eldin LM, Elsherbiny NM. Is serum level of methylated RASSF1A valuable in diagnosing hepatocellular carcinoma in patients with chronic viral hepatitis C? Arab J Gastroenterol. 2012; 13:111–5.

51. Miyoshi H, Fujie H, Moriya K, Shintani Y, Tsutsumi T, Makuuchi M, Kimura S, Koike K. Methylation status of suppressor of cytokine signaling-1 gene in hepatocellular carcinoma. J Gastroenterol. 2004; 39:563–9.

52. Chu PY, Yeh CM, Hsu NC, Chang YS, Chang JG, Yeh KT. Epigenetic alteration of the SOCS1 gene in hepatocellular carcinoma. Swiss Med Wkly. 2010; 140:w13065.

53. Saelee P, Chuensumran U, Wongkham S, Chariyalertsak S, Tiwawech D, Petmitr S. Hypermethylation of suppressor of cytokine signaling 1 in hepatocellular carcinoma patients. Asian Pac J Cancer Prev. 2012; 13:3489–93.

54. Zhang X, Wang J, Cheng J, Ding S, Li M, Sun S, Zhang L, Liu S, Chen X, Zhuang H, Lu F. An integrated analysis of SOCS1 down-regulation in HBV infection-related hepatocellular carcinoma. J Viral Hepat. 2014; 21:264–71.

55. Wong CM, Lee JM, Ching YP, Jin DY, Ng IO. Genetic and epigenetic alterations of DLC-1 gene in hepatocellular carcinoma. Cancer Res. 2003; 63:7646–51.

56. Ding S, Gong BD, Yu J, Gu J, Zhang HY, Shang ZB, Fei Q, Wang P, Zhu JD. Methylation profile of the promoter CpG islands of 14 “drug-resistance” genes in hepatocellular carcinoma. World J Gastroenterol. 2004; 10:3433–40.

57. Vivekanandan P, Torbenson M. Epigenetic instability is rare in fibrolamellar carcinomas but common in viral-associated hepatocellular carcinomas. Mod Pathol. 2008; 21:670–5.

58. Tchou JC, Lin X, Freije D, Isaacs WB, Brooks JD, Rashid A, De Marzo AM, Kanai Y, Hirohashi S, Nelson WG. GSTP1 CpG island DNA hypermethylation in hepatocellular carcinomas. Int J Oncol. 2000; 16:663–76.

59. Zhong S, Tang MW, Yeo W, Liu C, Lo YM, Johnson PJ. Silencing of GSTP1 gene by CpG island DNA hypermethylation in HBV-associated hepatocellular carcinomas. Clin Cancer Res. 2002; 8:1087–92.

60. Anzola M, Cuevas N, López-Martínez M, Saiz A, BurgosJJ, de Pancorbo MM. No association between GSTP1 gene aberrant promoter methylation and prognosis in surgically resected hepatocellular carcinoma patients from the Basque Country (Northern Spain). Liver Int. 2003; 23:249–54.

61. Wang J, Qin Y, Li B, Sun Z, Yang B. Detection of aberrant promoter methylation of GSTP1 in the tumor and serum of Chinese human primary hepatocellular carcinoma patients. Clin Biochem. 2006; 39:344–8.

62. Shih YL, Shyu RY, Hsieh CB, Lai HC, Liu KY, Chu TY, Lin YW. Promoter methylation of the secreted frizzled-related protein 1 gene SFRP1 is frequent in hepatocellular carcinoma. Cancer. 2006; 107:579–90.

63. Takagi H, Sasaki S, Suzuki H, Toyota M, Maruyama R, Nojima M, Yamamoto H, Omata M, Tokino T, Imai K, Shinomura Y. Frequent epigenetic inactivation of SFRP genes in hepatocellular carcinoma. J Gastroenterol. 2008; 43:378–89.

64. Xiao WH, Liu WW. Hemizygous deletion and hypermethylation of RUNX3 gene in hepatocellular carcinoma. World J Gastroenterol. 2004; 10:376–80.

65. Xiao WH, Liu WW. Analysis of methylation and loss of heterozygosity of RUNX3 gene in hepatocellular carcinoma and its clinical significance. Zhonghua Gan Zang Bing Za Zhi. 2004; 12:227–30.

66. Xiao WH, Liu WW. Improvement and application of methylation-specific polymerase chain reaction. Ai Zheng. 2004; 23:1354–6.

67. Mori T, Nomoto S, Koshikawa K, Fujii T, Sakai M, Nishikawa Y, Inoue S, Takeda S, Kaneko T, Nakao A: Decreased expression and frequent allelic inactivation of the RUNX3 gene at 1p36 in human hepatocellular carcinoma. Liver Int. 2005; 25:380–8.

68. Park WS, Cho YG, Kim CJ, Song JH, Lee YS, Kim SY, Nam SW, Lee SH, Yoo NJ, Lee JY. Hypermethylation of the RUNX3 gene in hepatocellular carcinoma. Exp Mol Med. 2005; 37:276–81.

69. Miyagawa K, Sakakura C, Nakashima S, Yoshikawa T, Kin S, Nakase Y, Ito K, Yamagishi H, Ida H, Yazumi S, Chiba T, Ito Y, Hagiwara A. Down-regulation of RUNX1, RUNX3 and CBFbeta in hepatocellular carcinomas in an early stage of hepatocarcinogenesis. Anticancer Res. 2006; 26:3633–43.

70. Fukai K, Yokosuka O, Chiba T, Hirasawa Y, Tada M, Imazeki F, Kataoka H, Saisho H. Hepatocyte growth factor

activator inhibitor 2/placental bikunin (HAI-2/PB) gene is frequently hypermethylated in human hepatocellular carcinoma. Cancer Res. 2003; 63:8674–9.

71. Tung EK, Wong CM, Yau TO, Lee JM, Ching YP, Ng IO. HAI-2 is epigenetically downregulated in human hepatocellular carcinoma, and its Kunitz domain type 1 is critical for anti-invasive functions. Int J Cancer. 2009; 124:1811–9.

72. Huang L, Li MX, Wang L, Li BK, Chen GH, He LR, Xu L, Yuan YF. Prognostic value of Wnt inhibitory factor-1 expression in hepatocellular carcinoma that is independent of gene methylation. Tumour Biol. 2011; 32:233–40.

73. Zhang C, Li H, Wang Y, Liu W, Zhang Q, Zhang T, Zhang X, Han B, Zhou G. Epigenetic inactivation of the tumor suppressor gene RIZ1 in hepatocellular carcinoma involves both DNA methylation and histone modifications. J Hepatol. 2010; 53:889–95.

74. Yu J, Zhang HY, Ma ZZ, Lu W, Wang YF, Zhu JD. Methylation profiling of twenty four genes and the concordant methylation behaviours of nineteen genes that may contribute to hepatocellular carcinogenesis. Cell Res. 2003; 13:319–33.

75. Poirier K, Chalas C, Tissier F, Couvert P, Mallet V, Carrié A, Marchio A, Sarli D, Gicquel C, Chaussade S, Beljord C, Chelly J, Kerjean A, et al. Loss of parental-specific methylation at the IGF2 locus in human hepatocellular carcinoma. J Pathol. 2003; 201:473–9.

76. Tang SH, Yang DH, Huang W, Zhou HK, Lu XH, Ye G. Hypomethylated P4 promoter induces expression of the insulin-like growth factor-II gene in hepatocellular carcinoma in a Chinese population. Clin Cancer Res. 2006; 12:4171–7.

77. Ruan XX, Wang SP, Chen XP, Ren PT. 5′CpG methylation of p14ARF gene in hepatocellular carcinoma. Chin J Gen Surg. 2007; 22:848–50.

78. Chen J, Yang C, Wu FX, Cao J. promoter methylation status and protein expression of p14ARF gene in hepatocellular carcinoma. J Diag Pathol. 2008; 15:202–5.

79. Wu FX, Chen J, Yang C, Cao J, Ou C, Zhao YN. p14ARF promoter methylation and its clinical correlation in hepatocellular carcinoma. J Guangxi Med Univ. 2009; 26:218–20.

80. Zhang JX, Wang Y, Zhao YP, Zhang B, Li J, Huang J. p14ARF promoter methylation and its clinical correlation in primary liver cancer in the population of North China. J Clin Hepatol. 2011; 27:1051–4.

81. Li HP, Gao B, Yu ZT, Liu JB, Zhang JC. Significance of detection of multigene methylation in liver cancer tissue for early diagnosis of liver cancer. J Clin Hepatol. 2013; 29:624–6.

82. Zhang JC, Yu ZT, Lv J, Xie F, Gao B, Li HP. The relationgship between Hepatitis B Virus infection and Multigene methylation in hepatocellular carcinoma. J Clin Intern Med. 2013; 30:163–5.

83. Huang JZ, Shen WL, Xia SS, Li B, Luo YG, Jiang HY. The status of pl6 gene in primary hepatocellularcarcinoma from a Chinese population. Ai Zheng. 2000; 19:45–7.

84. Liu JY, Qin Y, Sun ZL, Sun ZF. Methylation of p15 and p16 gene 5’ CpG island in human primary liver cancer. J Sichuan Univ. 2002; 39:127-32.

85. Chen XJ, Zhang HH, Zheng W, Li XX, Ye JX. p16 gene methylation status of and its effect on mRNA expression in hepatocellular carcinoma. Chin Oncol. 2005; 15:569–71.

86. Yang B. CDKN2A gene methylation and its clinical study in hepatocellular carcinoma. Nankai Univ. 2006.

87. Lin Q, Tang YM, Chen LB, Wang J. Analysis of promoter hypermethylation of p16gene,AFP and CEA in sera from primary liver cancer patients. J Dalian Med Univ. 2006; 28:290–2.

88. Zhang JC, Lv J, Li HP, Feng J, Wu JM, Hu LH. p16 promoter hypermethylation in the plasma DNA and its possible application in molecular diagnosis of hepatocellular carcinoma. Chin J Lab Med. 2006; 29:895–898.

89. Jiang L, Qin Y, Sun ZL, Sun ZF, Wang JH, Yang LC. Research on promoter region methylation status of tumor repressor gene p16 in human hepatocellular carcinoma by using methylation sensitive restriction enzyme and semi-nested PCR assay. J Sichuan Univ. 2007; 38:53–56.

90. Lou C. The experimental research on DNA methylation profile in hepatocelluIar carcinoma. Tianjin Med Univ 2008.

91. Zhu YZ. Research on Hepatitis B Virus X Protein promoting hypermethylation of p16INK4A promoter. Fudan Univ 2010.

92. He QF, Yan D, Wang LX. Promoter hypermethylation of RASSF1A and p16 gene in plasma of patients with hepatocellular carcinoma. Chin J Public Health. 2010; 26:819–21.

93. Wang FF. Correlation of aberrant methylation of p16, APC gene to MTHFR, TS genetic polymorphisms in hepatic carcinoma. Qingdao Univ 2011.

94. Chen YF. Circulating nucleic acids and DNA methylation in hepatocellular carcinoma. Sec Mil Med Univ. 2011.

95. Qi YP, Wang FF, Cui LH, Song Y. Detection of methylation of p16 and APC gene in liver-cancer tissue and plasma and its clinical significance. Med J Qilu. 2013; 28:189–91.

96. Li H. Study of aberrant methylation and clinical significanceof suppressor gene in hepatocellular carcinoma. Soochow Univ. 2013.

97. Yang JJ, Dang DM, Wang MQ, Ren CJ, Hui QY. The gene methylation of p16, Runx3 in patients with hepatocellular carcinoma. Modern Oncology. 2013; 21:1081–4.

98. Wang Z, Lv Y. Detection of DNA methylation and expression of DNMT1 mRNA in plasma of hepatocellular carcinoma. Chin J Public Health. 2014; 30:1233–4.

99. Wang YQ, Ye T, Liu JB. DNA methylation profile of tumor suppressor genes in diagnosis of primary hepatocellular carcinoma. Cancer Res Prev Treat. 2014; 41:1330–1334.

100. Dong XG, Guo WJ, Ding W. Detection of methylation of p16 and RASSF1A gene in plasma and tumor tissues from patientswith hepatocellular carcinoma and its clinical significance. Chin Med Biotechnol. 2016; 11:252–258.
101. Zhou XJ, Qin L, Xue WJ, Liu JX, Tian LP, Qian HX. Inactivation of RASSF1A gene in hepatocellular carcinoma. Chin J Hepatobiliary Surg. 2007; 13:351–2.
102. Qiu XQ, Chen G, Yu HP, Hu L. Detection of RASSF1A promoter hypermethylation in plasma of patients with primary hepatocellular carcinoma and its clinical significance. World Chin J Dig. 2009; 17:90–93.
103. He QF, Yan D, Wang LX. Promoter hypermethylation of RASSF1A and pl6 gene in plasma of patients with hepatocellular carcinoma. Chin J Public Health. 2010; 26:819–21.
104. Fei BJ, Huang ZH, Hua D, Hu Y, Cheng ZH, Yu J. Clinical significance of methylation of plasma Ras·association domain family 1A gene in the molecular diagnosis of hepatocellular carcinoma. Tumor. 2011; 31:742–7.
105. Zhang HJ. Prognostic significance of RASSFlA, APC, WIF-1 methylation in paraffin embedded tissues of HCC. Hebei Univ. 2011.
106. Gong HY, Liu ZG, Zhang HY. Detection of promoter hypermethylation of RASSF1A and CDH13 gene by nested methylation specific polymerase chain reaction in hepatocellular carcinoma patients. Life Science Res. 2011; 15:56–60.
107. Chen WS, Guo SL. Methylation status of RASSF1 gene promoter in primary hepatocellular carcinoma and its clinical applications. Chin J Modern Med. 2011; 21:1864–7.
108. Naglaa IA, Heba MA, Tawheed M, Hanan FF, Awad ME. Blood Ras-Association Domain Family 1 A gene methylation status in some liver diseases. Life Science J. 2011; 8:531–9.
109. Xue WJ, Feng Y, Li P, Mao QS, Guan HG, Qian HX. Correlation analysis of ras association domain family IA gene methylation and environmental factors to hepatocellular carcinoma. Chin J Exp Surg. 2012; 29:650–2.
110. Zhang YY, Jiang Q, Chen SQ, Gu RM, Zhang XM, Zhu M, Ma CJ. Effects of DNA methylation and micronucleus formation on pathogenesis of hepatocellular carcinoma by methylation-specific polymerase chain reaction and microscopy. Chin J Biomed Eng. 2013; 19:11–5.
111. Zhao HJ, Li SM, Zhang HJ, Guo ZJ, Fan HY, Yang GR, Wang L, Zhang N. Diagnostic and prognostic value of RASSF1A and APC gene methylation in plasma of liver cancer patients. Chin J Cancer Prev Treat. 2013; 20:53–7.
112. Fan HY, Zhang HJ, Guo ZJ, Li SM. Prognostic significance of RASSF1 A and W IF-1 methylation in the cancer tissues of patients with HCC. J Shandong Univ. 2013; 51:89–93.
113. Chen TG, Li JG, Lin ZC. Promoter methylation of RASSFlA gene in hepatocellular carcinoma and its clinical significance. Chin J Gen Surg. 2013; 28:300–3.
114. Li YN, Wang YQ. The study of Golgi protein 73 and RASSF1A promoter hypermethylation in the diagnosis of hepatocellular carcinoma. Chin J Health Lab Tec. 2014; 24:3443–6.
115. Liu DS, Qian HG, Shen G, Chen YM. Detection of RASSF1A promoter hypermethylation in serum of the patients with hepatocellular carcinoma and its clinical significance. Chin J Health Lab Tec. 2015; 25:3300–2.
116. Liu DS, Chen YM, Dong XY, Zhang WY, Yu DJ. Promoter methylation of multiple genes for diagnosis of HBV-related hepatocellular carcinoma. Chin J Modern Med. 2016; 26:45–9.
117. Liu JB, Zhang SQ, Shi MX, Shao BF, Zhang YX. Relationship between methylation status of DLC-1 gene and metastasis of hepatocellular carcinoma. World Chin J Dig. 2008; 16:1237–40.
118. Liu JB. The clinical value of folate status and gene methylation in hepatocellular carcinoma and esophageal carcinoma. Jiangsu Univ 2012.
119. Wang BS, Zhang DG, Zhang ML, Dai XW, Yu LL. Detection of promoter methylation of CDH1 gene in hepatocellular carcinoma patients and its clinical significance. Chin J Gerontol. 2006; 26:926–8.
120. Zhang YC, Chen JX, Gong L, Jiang Yu, Chen YP. Expression of E-cadherin gene and methylation of promoter in hepatocellular carcinoma. J Pract Oncol. 2008; 23:314–7.
121. Huang WQ, Yang WL, Chai XJ, Chen KF, Wei L, Li B, Qin Y. Aberrant promoter CpG islands methylation of E-cadherin in human primary hepatocellular carcinomas. Chin J Bases Clin General Surg. 2011; 18:514–9.
122. Sheng D. Methylation profile of the promoter CpG islands of fourteen “drug-resistance” genes in hepatocellular carcinoma. Fudan Univ. 2004.
123. Yang WF. Detection aberrant promoter methylation of GSTP1 gene in hepatocellular carcinoma. Soochow Univ. 2004.
124. Liu WJ, Wang L, Wang JP, Li JQ, Zhang CQ, Zheng L, Yuan YF. Correlations of CpG island methylator phenotype and OPCML gene methylation to carcinogenesis of hepatocellular carcinoma. Chin J Cancer. 2006; 25:696–700.
125. Zhang YC, Chen YP, Chen JX, Dong PH, Deng CS. Study on the polymorphisms and promoter methylation and expression of the glutathione Stransferases P1 gene in hepatocellular carcinoma. Chin J Dig. 2006; 26:468–72.
126. Wu L, Qian YB, Zhu LX, Geng XP, Xiong QR. Promoter methylation and mRNA expression of SFRP1 and APC gene in hepatocellular carcinoma. Chin J Gen Surg. 2008; 17:29–33.
127. Su Q. Clinical research of the methylation status of 14-3-3 sigma gene and SFRP genes in hepatitis B virus-related hepatocellular carcinoma. Anhui Med Univ. 2009.
128. Zhang HY, Li K, He XB. Methylation status analysis of Runx3 gene in hepatocellular carcinoma and clinical significance. Clinical Focus. 2009; 24:409–12.
129. Zhang HY, Liu J, Xie TB. Promoter hypermethylation of the Runx3 gene in hepatocellular carcinoma. Acta Med Univ Sci Technol Huazhong. 2009; 38:23–6.
130. Jiang XJ, Li JG, Lin ZC. Relationship between promoter methylation and mRNA expression of RUNX3 gene

in human primary hepatocellular carcinoma and its significance. Chin J Exp Surg. 2011; 28:1640–2.

131. Li JG, Jiang XJ. Methylation of RUNX3 gene promoter in HCC and its significance. J Third Mil Med Univ. 2012; 34:1933–5.

132. Liang H. Frequent epigenetic inactivation of Wnt inhibitory factor-1 in hepatocellular carcinoma. Sun Yatsen University 2008.

133. Ding Z, Qian YB, Xiong QR, Yu HZ, Li FY, Zhang C, Xu YC. Promoter methylation and mRNA expression of DKK-3 gene in hepatocellular carcinoma. J Clin Med Pract. 2009; 13:12–5.

134. Tong B. Methylation of WIF-1 gene and its expression in hepatocellular carcinoma. Tianjin Med Univ. 2010.

135. Liu Y, Zhao Y, Zhang XS, Liu JB, Mao GX. Clinical value of the expression of the WIF-1 in plasma and tissue from hepatocellular carcinoma (HCC) patients. J Mod Lab Med. 2011; 26:63–6.

136. Liu Y, Liu F, Shen F, Mao GX, Yuan XL. Expression and clinical significance of WIF-1 DNA methylation in hepatic cancer. J Clin Pathol Res. 2015; 35:1943–7.

137. Qian B, Zhu LX, Geng XP, Xiong QR, Qian YB, Li XM. Methylation of MGMT, DAPK, THBS1 and RIZ1 genes in hepatocellular carcinoma. Chin J Gen Surg. 2005; 20:291–4.

138. Huang W, Yang DH, Tang SH. Methylation of IGF-II P4 gene promoter in hepatocellular carcinoma and its significance. Guangdong Med J. 2005; 26:782–4.

139. Tang SH, Yang DH, Huang W, Zhou HK, Lu YH, Ye G. Methylation of insulin-like growth factor-II P4 gene promoter in hepatocellular carcinoma tissues and its significance. Natl Med J China. 2006; 86:565–7.

140. Qian J, Shen YC, Qiu LW, Yao NH, Yao DF. Methylational status of insulin-like growth factor-II gene promoter and pathological characteristics in hepatocellular carcinoma. Med J of Communications. 2010; 24:112–5.

141. Dong ZZ, Yao DF, Wu W, Qiu LW, Yao NH, Yan XD, Yu DD, Chen J. Correlation between epigenetic alterations in the insulin growth factor-II gene and hepatocellular carcinoma. Chin J Hepatol. 2012; 20:593–7.

142. Yuan WP, Wu FX, Huang S, Yang C, Liu JY, Zhao YN. Promoter methylation status and protein expression of APC gene in hepatocellular carcinoma. Modern Oncology. 2010; 18:1795–7.

143. Li MX. DNA hypermethylation leads to silencing of OPCML gene in hepatocellular carcinoma. Sun Yatsen Univ. 2007.

144. Huang J, Zhen L, Wang L, Wang JP, Li JQ, Zhang CQ, Yuan YF. The association of hMLH1 gene methylation and curative effect of adjuvant TACE after hepatectomy for hepatocellular carcinoma. J Sun Yatsen Univ. 2006; 27:3–5.

SOCS1

Study or Subgroup	Carcinoma tissues Events	Total	Adjacent tissues Events	Total	Weight	Odds Ratio M-H, Random, 95% CI	Year
**China**							
Liu Wenji 2006	39	50	24	48	18.1%	3.55 [1.48, 8.52]	2006
Lou Cheng 2009	35	60	32	60	20.2%	1.23 [0.60, 2.52]	2009
Peiyi Chu 2010	18	46	19	46	18.7%	0.91 [0.40, 2.10]	2010
Binkui Li 2010	95	115	24	48	19.9%	4.75 [2.26, 9.99]	2010
X Zhang 2014	65	116	63	116	23.1%	1.07 [0.64, 1.80]	2014
**Total (95% CI)**		**387**		**318**	**100.0%**	**1.79 [0.94, 3.40]**	
Total events	252		162				
Heterogeneity: $Tau^2 = 0.40$; $Chi^2 = 15.94$, df = 4 (P = 0.003); $I^2 = 75\%$							
Test for overall effect: Z = 1.76 (P = 0.08)							
**Japan**							
Osamu Okochi 2003	30	50	0	50	16.1%	150.27 [8.77, 2575.00]	2003
Hideyuki Miyoshi 2004	9	22	1	22	18.6%	14.54 [1.65, 128.44]	2004
S Nomoto 2007	38	74	2	51	21.0%	25.86 [5.85, 114.24]	2007
Naoshi Nishida 2008	48	75	53	77	23.1%	0.81 [0.41, 1.58]	2008
Eric J Formeister 2010	39	43	41	45	21.1%	0.95 [0.22, 4.07]	2010
**Total (95% CI)**		**264**		**245**	**100.0%**	**6.89 [0.92, 51.81]**	
Total events	164		97				
Heterogeneity: $Tau^2 = 4.47$; $Chi^2 = 36.96$, df = 4 (P < 0.00001); $I^2 = 89\%$							
Test for overall effect: Z = 1.87 (P = 0.06)							

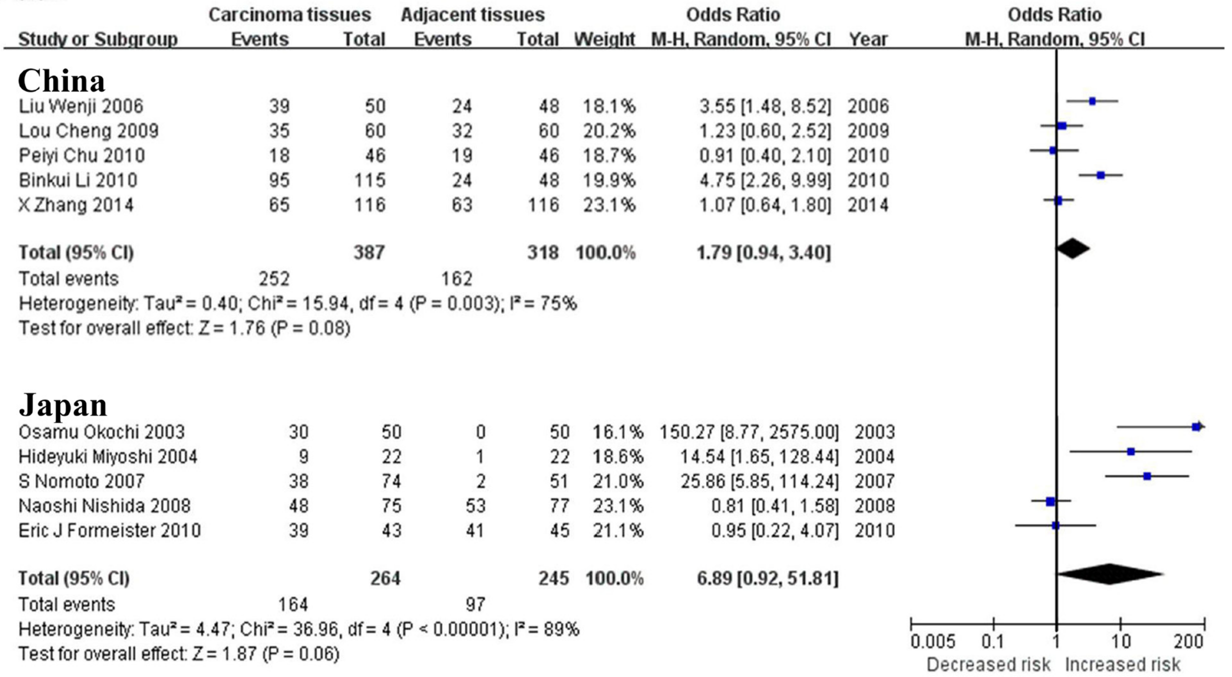

Supplementary Figure S1: Forest plots of *SOCS1* methylation between HCC tumor tissues and adjacent tissues in the meta-analysis.

Supplementary Table S1: Analysis of heterogeneity sources of 24 aberrant methylated genes between HCC tumor tissues and adjacent tissues in geographical populations. See Supplementary_Table_S1

Supplementary Table S2: Analysis of heterogeneity sources of 17 aberrant methylated genes between HCC tumor tissues and normal tissues in geographical populations. See Supplementary_Table_S2

Supplementary Table S3: Analysis of heterogeneity sources of six aberrant methylated genes between HCC tumor serums and normal serums in geographical populations

Gene	Geographical population	Studies (n)	Coefficient	95% CI	*P* value
RASSF1A	China	14	0.5639	[-3.3362, 4.464]	0.756
	Egypt	1	1.29449	[-2.76, 5.349]	0.497
	Singapore	1	—	—	—
p16	China	8	−2.6813	[−8.585, 3.222]	0.296
	Singapore	1	—	—	—
CDH1	China	2	—	—	—
	Singapore	1	—	—	—
RUNX3	China	2	—	—	—
	Singapore	1	—	—	—
GSTP1	China	3	—	—	—
WIF1	China	3	—	—	—

Analysis of heterogeneity sources of RASSF1A in Singapore, p16 in Singapore, CDH1 in China and Singapore, RUNX3 in China and Singapore, GSTP1 in China and WIF1 in China was not applicable, because the data of these genes were insufficient.
