# Supplementary material for: Meta-analysis of DNA methylation biomarkers in hepatocellular carcinoma
Source: Oncotarget. 2016 Nov 8;7(49):81255–67. doi: 10.18632/oncotarget.13221 (PMC5348390; doi:10.18632/oncotarget.13221)
Supplement: Supplementary file 2 [file oncotarget-07-81255-s002.doc]

Supplementary Table 1 Analysis of heterogeneity sources of 24 aberrant methylated genes between HCC tumor tissues and adjacent tissues in geographical populations

| Gene | Geographical population | Studies (n) | Coefficient | 95% CI | P value |
| --- | --- | --- | --- | --- | --- |
| *p16* | China | 28 | 0.133 | [-1.874, 2.142] | 0.893 |
|  | Japan | 6 | 0.659 | [-1.523, 2.84] | 0.544 |
|  | Germany | 3 | 0.257 | [-2.245, 2.759] | 0.836 |
|  | Korea | 2 | 0.920 | [-1.932, 3.77] | 0.517 |
|  | America | 1 | -0.595 | [-4.263, 3.072] | 0.744 |
|  | Egypt | 1 | -2.131 | [-0.15, 5.069] | 0.806 |
|  | Australia and South Africa | 1 | 0.307 | [-1.874, 2.142] | 0.893 |
|  | Spain | 1 | — | — | — |
| *RASSF1A* | China | 20 | -0.59 | [-4.136, 2.956] | 0.733 |
|  | Japan | 3 | -0.0827 | [-3.824, 3.659] | 0.964 |
|  | Korea | 2 | 0.731 | [-3.258, 4.712] | 0.708 |
|  | China and America | 1 | -0.756 | [-4.584, 3.071] | 0.686 |
|  | Germany | 1 | — | — | — |
|  | Egypt | 1 | — | — | — |
| *GSTP1* | China | 12 | -1.038113 | [-2.694745, 0.6185184] | 0.203 |
|  | Japan | 5 | -0.0075692 | [-1.75738, 1.742241] | 0.993 |
|  | Germany | 1 | -1.353504 | [-3.797767, 1.090758] | 0.258 |
|  | America | 1 | 0.1272213 | [-2.724841, 2.979284] | 0.926 |
|  | Spain | 1 | — | — | — |
| *p14* | China | 11 | 0.424 | [-3.61, 4.46] | 0.82 |
|  | America | 1 | -0.06 | [-6.322, 6.202] | 0.98 |
|  | Germany | 1 | 1.854 | [-4.329, 8.038] | 0.515 |
|  | Egypt | 1 | -0.798 | [-6.352, 4.755] | 0.752 |
|  | Australia and South Africa | 1 | -0.438 | [-5.711, 4.83] | 0.855 |
|  | Japan | 2 | — | — | — |
| *APC* | China | 9 | -0.1357308 | [-2.846965, 2.575503] | 0.913 |
|  | Japan | 3 | 0.564448 | [-2.301495, 3.430391] | 0.67 |
|  | America | 1 | 0.1665733 | [-3.416103, 3.749249] | 0.92 |
|  | Egypt | 1 | -1.874236 | [-5.151105, 1.402633] | 0.231 |
|  | Germany | 1 | — | — | — |
| *RUNX3* | China | 9 | -0.0192883 | [-3.514698, 3.476121] | 0.99 |
|  | Japan | 3 | -0.3201285 | [-4.129155, 3.488898] | 0.855 |
|  | Korea | 1 | — | — | — |
| *SOCS1* | China | 5 | -1.419 | [-5.654, 2.814] | 0.462 |
|  | Japan | 5 | -0.292 | [-4.611, 4.027] | 0.88 |
|  | America | 1 | 1.186 | [-4.575, 6.947] | 0.648 |
|  | China and America | 1 | -0.17 | [-5.321, 4.98] | 0.941 |
|  | Germany | 1 | — | — | — |
| *CDH1* | China | 7 | -0.9232629 | [-4.782827, 2.936301] | 0.566 |
|  | Japan | 1 | -1.085615 | [-5.94372, 3.77249] | 0.591 |
|  | America | 1 | 0.7004465 | [-4.817308, 6.218201] | 0.757 |
|  | Egypt | 1 | -1.43697 | [-6.389983, 3.516043] | 0.489 |
|  | Australia and South Africa | 1 | -2.534281 | [-7.421481, 2.35292] | 0.24 |
|  | Korea | 1 | — | — | — |
| *p15* | China | 7 | -1.347358 | [-7.425151, 4.730436] | 0.593 |
|  | Japan | 1 | -2.010449 | [-8.733375, 4.712478] | 0.477 |
|  | America | 1 | 1.220715 | [-7.098641, 9.54007] | 0.722 |
|  | Egypt | 1 | -2.031068 | [-8.636802, 4.574666] | 0.465 |
|  | Australia and South Africa | 1 | -1.89812 | [-8.092219, 4.29598] | 0.467 |
|  | Korea | 1 | — | — | — |
| *MGMT* | China | 6 | 0.6806881 | [-2.397738, 3.759114] | 0.617 |
|  | Japan | 2 | -1.315424 | [-5.58945, 2.958602] | 0.49 |
|  | Egypt | 1 | — | — | — |
|  | Australia and South Africa | 1 | — | — | — |
| *PRDM2* | China | 5 | 0.2504585 | [-1.845237, 2.346154] | 0.786 |
|  | Japan | 3 | 1.141096 | [-1.109623, 3.391815] | 0.270 |
|  | Australia and South Africa | 1 | — | — | — |
| *WIF1* | China | 8 | — | — | — |
| *DAPK1* | China | 4 | 1.312646 | [-5.495458, 8.12075] | 0.494 |
|  | Egypt | 1 | 0.9399057 | [-6.49792, 8.377732] | 0.641 |
|  | Japan | 1 | — | — | — |
| *SFRP1* | Japan | 1 | -0.9600963 | [-3.559221, 1.639029] | 0.325 |
|  | China | 4 | — | — | — |
| *RARβ* | China | 3 | — | — | — |
|  | America | 1 | — | — | — |
|  | Egypt | 1 | — | — | — |
| *IGF2* | China | 5 | — | — | — |
| *p73* | China | 3 | 1.591443 | [-7.218081, 10.40097] | 0.518 |
|  | Egypt | 1 | -0.145889 | [-9.699367, 9.407589] | 0.954 |
|  | America | 1 | — | — | — |
| *hMLH1* | China | 4 | — | — | — |
| *DLC1* | China | 3 | — | — | — |
| *p53* | China | 2 | — | — | — |
|  | Korea | 1 | — | — | — |
| *SPINT2* | China | 2 | — | — | — |
|  | Japan | 1 | — | — | — |
| *RB1* | China | 3 | — | — | — |
| *OPCML* | China | 3 | — | — | — |
| *WT1* | China | 3 | — | — | — |

Analysis of heterogeneity sources of *p16* in Spain, *RASSF1A* in Germany and Egypt, *GSTP1* in Spain, *p14* in Japan, *APC* in Germany, *RUNX3* in Korea, *SOCS1* in Germany, *CDH1* in Korea, *p15* in Korea, *MGMT* in Egypt, Australia and South Africa, *PRDM2* in Australia and South Africa, *WIF1* in China, *DAPK1* in Japan, *SFRP1* in China, *RARβ* in China, America and Egypt, *IGF2* in China, *p73* in America, *hMLH1* in China, *DLC1* in China, *p53* in China and Korea, *SPINT2* in China and Japan, *RB1* in China, *OPCML* in China and *WT1* in China was not applicable, because the data of these genes were insufficient.
