# Supplementary material for: Meta-analysis of DNA methylation biomarkers in hepatocellular carcinoma
Source: Oncotarget. 2016 Nov 8;7(49):81255–67. doi: 10.18632/oncotarget.13221 (PMC5348390; doi:10.18632/oncotarget.13221)
Supplement: Supplementary file 3 [file oncotarget-07-81255-s003.doc]

Supplementary Table 2 Analysis of heterogeneity sources of 17 aberrant methylated genes between HCC tumor tissues and normal tissues in geographical populations

| Gene | Geographical population | Studies (n) | Coefficient | 95% CI | P value |
| --- | --- | --- | --- | --- | --- |
| *p16* | China | 15 | -0.9685312 | [-4.157728, 2.220666] | 0.536 |
|  | Japan | 6 | -1.763947 | [-4.964881, 1.436986] | 0.266 |
|  | Germany | 3 | -0.4982309 | [-4.017173,3.020711] | 0.772 |
|  | Korea | 2 | -0.7777919 | [-4.567462, 3.011878] | 0.675 |
|  | America | 2 | -1.50266 | [-5.158365, 2.153045] | 0.404 |
|  | Egypt | 1 | -2.701185 | [-6.173906, 0.7715368] | 0.121 |
|  | Australia and  South Africa | 1 | -2.847229 | [-6.262409, 0.5679511] | 0.098 |
|  | France | 1 | — | — | — |
| *RASSF1A* | China | 10 | 0.5757033 | [-1.372559, 2.523965] | 0.536 |
|  | Japan | 2 | -2.165307 | [-4.229379, 0.1012344] | 0.041 |
|  | Korea | 2 | -.2507445 | [-2.829726, 2.328237] | 0.838 |
|  | Germany | 2 | 1.271098 | [-2.082076, 4.624273] | 0.430 |
|  | America | 2 | -1.587975 | [-4.168457, 0.9925071] | 0.208 |
|  | Egypt | 1 | 3.446906 | [-1.236208, 8.130019] | 0.137 |
|  | China and America | 1 | -0.3366342 | [-3.932699, 3.259431] | 0.844 |
|  | Thailand | 1 | — | — | — |
| *APC* | China | 6 | -1.493035 | [-6.517959, 3.531889] | 0.505 |
|  | Japan | 3 | -2.96651 | [-2.96651, 2.182613] | 0.215 |
|  | Germany | 2 | -0.4910665 | [-6.890773, 5.90864] | 0.861 |
|  | America | 1 | -3.856392 | [-9.632269, 1.919485] | 0.158 |
|  | Egypt | 1 | -4.68769 | [-10.5548, 1.179416] | 0.101 |
|  | Korea | 1 | — | — | — |
| *GSTP1* | China | 6 | -1.524722 | [-5.094296, 2.044851] | 0.353 |
|  | Japan | 2 | -3.41927 | [-6.985926, 0.1473858] | 0.058 |
|  | Germany | 2 | -1.267958 | [-6.057855, 3.521939] | 0.559 |
|  | America | 2 | -4.385558 | [-7.930558, -0.840558] | 0.021 |
|  | Korea | 1 | — | — | — |
| *CDH1* | China | 3 | 1.466061 | [-3.747945, 6.680067] | 0.35 |
|  | Korea | 2 | 2.016703 | [-2.747649, 6.781055] | 0.210 |
|  | America | 1 | -2.443206 | [-9.56808, 4.681669] | 0.278 |
|  | Egypt | 1 | 0.1352134 | [-3.999037, 4.269464] | 0.901 |
|  | Australia and  South Africa | 1 | -0.2767811 | [-4.596458, 4.042895] | 0.809 |
|  | Germany | 1 | -1.339876 | [-9.077168, 6.397415] | 0.534 |
|  | Japan | 1 | — | — | — |
| *p15* | China | 5 | 2.461808 | [-2.179234, 7.102849] | 0.190 |
|  | America | 1 | 0.8397507 | [-4.685945, 6.365446] | 0.662 |
|  | Australia and  South Africa | 1 | 1.884217 | [-2.531234, 6.299667] | 0.268 |
|  | Egypt | 1 | 1.924111 | [-4.31144, 8.159661] | 0.399 |
|  | Korea | 1 | — | — | — |
| *RUNX3* | China | 4 | -0.8734831 | [-3.548224, 1.801258] | 0.439 |
|  | America | 1 | -1.475934 | [-5.739688, 2.787819] | 0.414 |
|  | Japan | 3 | — | — | — |
| *SOCS1* | China | 2 | -1.380157 | [-11.58, 8.819687] | 0.619 |
|  | Japan | 2 | -2.396283 | [-12.43439, 7.641827] | 0.412 |
|  | America | 1 | -4.468557 | [-15.90853, 6.97142] | 0.235 |
|  | Germany | 1 | -2.022871 | [-13.16847, 9.122724] | 0.517 |
|  | China and America | 1 | 0.2646928 | [-11.98706, 12.51645] | 0.934 |
|  | Thailand | 1 | — | — | — |
| *MGMT* | China | 4 | 2.450991 | [-2.518249, 7.420231] | 0.243 |
|  | Egypt | 1 | 4.348987 | [-1.830234, 10.52821] | 0.122 |
|  | Japan | 2 | — | — | — |
|  | Australia and  South Africa | 1 | — | — | — |
| *PRDM2* | China | 4 | -0.3530406 | [-2.672849, 1.966768] | 0.722 |
|  | Australia and  South Africa | 1 | -1.601567 | [-4.679857, 1.476723] | 0.25 |
|  | Japan | 3 | — | — | — |
| *SFRP1* | China | 4 | -0.0338935 | [-3.14855, 3.080763] | 0.977 |
|  | America | 1 | -0.751928 | [-5.635485, 4.131629] | 0.691 |
|  | Japan | 2 | — | — | — |
| *DAPK1* | China | 4 | 0.1557242 | [-6.656158, 6.967607] | 0.931 |
|  | Japan | 1 | -1.923646 | [-11.50455, 7.657262] | 0.479 |
|  | Egypt | 1 | 0.3587365 | [-6.843457, 7.56093] | 0.85 |
|  | Korea | 1 | — | — | — |
| *p14* | China | 3 | — | — | — |
|  | America | 1 | — | — | — |
|  | Egypt | 1 | — | — | — |
|  | Australia and  South Africa | 1 | — | — | — |
| *RARβ* | China | 2 | — | — | — |
|  | America | 1 | — | — | — |
|  | Germany | 1 | — | — | — |
|  | Egypt | 1 | — | — | — |
| *IGF2* | China | 5 | — | — | — |
| *hMLH1* | China | 3 | — | — | — |
| *p73* | China | 2 | — | — | — |
|  | Egypt | 1 | — | — | — |

Analysis of heterogeneity sources of *p16* in France, *RASSF1A* in Thailand, *APC* in Korea, *GSTP1* in Korea, *CDH1* in Japan, *p15* in Korea, *RUNX3* in Japan, *SOCS1* in Thailand, *MGMT* in Japan, Australia and South Africa, *PRDM2* in Japan, *SFRP1* in Japan, *DAPK1* in Korea, *p14* in China, America, Egypt, Australia and South Africa, *RARβ* in China, America, Germany and Egypt, *IGF2* in China, *hMLH1* in China and *p73* in China and Egypt was not applicable, because the data of these genes were insufficient.
